# Cytotoxic sigma-2 ligands trigger cancer cell death via cholesterol-induced-ER-stress

**DOI:** 10.1038/s41419-024-06693-8

**Published:** 2024-05-02

**Authors:** Rony Takchi, Bethany C. Prudner, Qingqing Gong, Takaomi Hagi, Kenneth F. Newcomer, Linda X. Jin, Suwanna Vangveravong, Brian A. Van Tine, William G. Hawkins, Dirk Spitzer

**Affiliations:** 1 Department of Surgery, Washington University School of Medicine, St. Louis, MO USA; 2 Department of Medical Oncology, Washington University School of Medicine, St. Louis, MO USA; 3 Department of Pediatric Hematology/Oncology, St. Louis Children’s Hospital, St. Louis, MO USA; 4 Alvin J Siteman Cancer Center, Barnes-Jewish Hospital and Washington University School of Medicine, St. Louis, MO USA

**Keywords:** Biological sciences, Drug delivery

## Abstract

Sigma-2-ligands (S2L) are characterized by high binding affinities to their cognate sigma-2 receptor, overexpressed in rapidly proliferating tumor cells. As such, S2L were developed as imaging probes (ISO1) or as cancer therapeutics, alone (SV119 [C6], SW43 [C10]) and as delivery vehicles for cytotoxic drug cargoes (C6-Erastin, C10-SMAC). However, the exact mechanism of S2L-induced cytotoxicity remains to be fully elucidated. A series of high-affinity S2L were evaluated regarding their cytotoxicity profiles across cancer cell lines. While C6 and C10 displayed distinct cytotoxicities, C0 and ISO1 were essentially non-toxic. Confocal microscopy and lipidomics analysis in cellular and mouse models revealed that C10 induced increases in intralysosomal free cholesterol and in cholesterol esters, suggestive of unaltered intracellular cholesterol trafficking. Cytotoxicity was caused by cholesterol excess, a phenomenon that contrasts the effects of NPC1 inhibition. RNA-sequencing revealed gene clusters involved in cholesterol homeostasis and ER stress response exclusively by cytotoxic S2L. ER stress markers were confirmed by qPCR and their targeted modulation inhibited or enhanced cytotoxicity of C10 in a predicted manner. Moreover, C10 increased sterol regulatory element-binding protein 2 (SREBP2) and low-density lipoprotein receptor (LDLR), both found to be pro-survival factors activated by ER stress. Furthermore, inhibition of downstream processes of the adaptive response to S2L with simvastatin resulted in synergistic treatment outcomes in combination with C10. Of note, the S2L conjugates retained the ER stress response of the parental ligands, indicative of cholesterol homeostasis being involved in the overall cytotoxicity of the drug conjugates. Based on these findings, we conclude that S2L-mediated cell death is due to free cholesterol accumulation that leads to ER stress. Consequently, the cytotoxic profiles of S2L drug conjugates are proposed to be enhanced via concurrent ER stress inducers or simvastatin, strategies that could be instrumental on the path toward tumor eradication.

## Introduction

The sigma-2 receptor (S2R) is overexpressed on rapidly proliferating tumor cells relative to non-transformed host tissues [1–3]. Initially, S2L were successfully employed as tumor-homing imaging probes able to identify solid tumors [4]. Later on, a family of structurally diverse, high-affinity, sigma-2 ligands (S2L) were found to rapidly internalize into cancer cells, enabling the design of cancer-selective therapeutics that can achieve maximum treatment benefits while minimizing systemic toxicities [5, 6]. Consequently, S2L were chemically linked to fluorophores (SW120 [C10-NBD]) to study S2L uptake characteristics/kinetics [7], or to cytotoxic payloads to perform cancer-selective drug delivery, resulting in substantial increases in potency in vitro and in preclinical mouse models of cancer [8–12]. The S2R was identified as transmembrane protein 97 (TMEM97) [13], reported to be involved in cholesterol homeostasis [14–17].

Cholesterol is a crucial component of the plasma membrane as it maintains its stability and fluidity and creates lipid rafts implicated in signal transduction, apoptosis, and metastasis [18, 19]. However, excess of intracellular free cholesterol is cytotoxic [20]. Consequently, intracellular cholesterol levels require tight regulation mediated by an intricate feedback mechanism [21, 22], involving sterol regulatory element-binding protein 2 (SREBP2), the central transcriptional regulator of cholesterol homeostasis. More specifically, SREBP2 is an ER-anchored precursor that binds to SREBP-cleavage activating protein (SCAP) which in turn senses and responds to fluctuations in ER cholesterol levels. When ER membrane cholesterol is depleted, conformational changes in SCAP induces the translocation of the SCAP–SREBP2 complex from the ER to the Golgi for proteolytic cleavage/activation of SREBP2. Upon nuclear translocation, cleaved SREBP2 binds to the sterol regulatory element (SRE) sequence in the promoter of target genes and upregulates cholesterologenic genes including 3-hydroxy-3-methyl-glutaryl coenzyme A reductase (HMGCR), the rate-limiting enzyme for de novo cholesterol synthesis, and low-density lipoprotein receptor (LDLR), the key import mechanism of dietary cholesterol complexed to LDL [14, 17, 21, 22]. Moreover, a pharmacological inhibitor of NPC1 (U18666a) traps free cholesterol in the lysosomes preventing its transport to the ER and therefore is predicted to activate SREBP2 and subsequently LDLR [23].

On the other hand, elevated levels of free cholesterol induce a series of negative feedback mechanisms ranging from preventing the activation of SREBP2, to rapid esterification via acyl-CoA cholesterol acyltransferase-1 (ACAT-1) and expulsion from the cell via ATP binding cassette A1 (ABCA1) in order to protect the cells from cholesterol-mediated cytotoxicity [20, 22]. Additionally, excess cholesterol induces the ER stress response which activates the transcription of several genes involved in both survival and apoptosis [24]. Particularly, ER stress can be triggered by the accumulation of excessive levels of cholesterol in the ER, and the subsequent ER stress response results in (1) attenuation of protein translation, (2) the activation of ER-associated degradation (ERAD)/autophagy to eliminate the misfolded proteins, (3) the proliferation of the ER compartment which requires lipids and phospholipids downstream of SREBP2 necessary for production of ER membrane, and (4) the activation of both caspase-dependent and caspase-independent apoptosis if stress persists and homeostasis cannot be restored [25–27].

The aim of this study was to revisit the mechanism of S2L-mediated cytotoxicity, in the context of the recently discovered role of S2R in regulating cholesterol homeostasis. We found that C10 increases intracellular free cholesterol which causes ER stress which in turn initiates the ER stress response and activates SREBP2, despite the excess cholesterol, as one of the adaptive mechanisms to maintain homeostasis and prevent cell death. Abrogating this adaptive mechanism directly with fatostatin, or indirectly with simvastatin potentiates C10 cytotoxicity. Remarkably, the two functionally distinct S2L-based drug conjugates, C10-SMAC (SW IV-134) and C6-Erastin (ACXT-3102), retained the phenotypic properties of their respective delivery vehicles SW43 (C10) and SV119 (C6), respectively, with regard to cholesterol-induced ER stress.

## Methods

The list of resources used in this study are summarized in Table S1. OVCAR-8 and SYO-1 were generous gifts from Dr. Katherine C. Fuh [28] and Dr. Brian A. Van Tine [29] respectively.

### Cell culture

AsPC-1 and OVCAR-8 were cultured in RPMI supplemented with 1% Antibiotic-Antimycotic (AA) and either 10% or 15% FBS respectively. HPAC, MIAPaCa-2, and SYO-1 were cultured in DMEM supplemented with 10% FBS, and 1% AA, while KP2 were cultured in 1:1 mixture DMEM:F12 with 10% FBS, and 1% AA. All cells were cultured at 37 °C in humidified incubator with 5% CO_2_ and low passage number was maintained. Cells were also cultured in prophylactic plasmocin and were regularly tested and verified to be mycoplasma negative.

### Proliferation and cell death assays

IncuCyte ZOOM time-lapse microscopy was used to monitor red nuclear fluorescence and green yoyo-1 fluorescence that quantified proliferation and cell-death signals respectively. NucRed+ cells were seeded in 96-well plate at 5000–7000 cells per 100 µl media. The detailed methodology of generation of NucRed+ cell lines and analysis of Incucyte results can be found in (Additional file 1).

### Synergy determination

Following the Chou–Talalay method [30], IC50 values for C10 and simvastatin were first assessed in AsPC-1 and HPAC cell lines using Incucyte. In a 96-well plate, cells were plated at 5000 and 7000 cells/well respectively, then incubated at 37 °C for 24–36 h to allow adhesion. Cells were then treated with serial dilutions of C10, simvastatin, or a combination of both. Both drugs were combined at a constant C10-to-simvastatin ratio. Viability data were generated using the Proliferation IncuCyte assay as described above. CompuSyn software v1.0 was used to generate CI values; synergy was defined as a CI < 0.9.

### Cell viability assay

Cell viability was evaluated using CellTiterGlo 2.0 reagent according to the manufacturer’s protocol. Results were normalized to control as ${{\rm{Death}}}\left( \% \right)=1-{{\rm{Viability}}}\left( \% \right)=\left[1-\frac{{{{\rm{Absorbance}}}}_{{{\rm{treated}}}}}{{{{\rm{Absorbance}}}}_{{{\rm{control}}}}}\times 100\right]$.

### Flow cytometry

Cells were plated at 300,000–500,000 per 2 ml media in 6-well plates for 24 h. After washing with PBS, and addition of fresh serum-free media, cells were treated with 100 nM C10-NBD for 30 min at 37 °C or 4 °C to study if uptake is energy dependent [31]. Alternatively, cells were pretreated with 25 µM Pitstop 2 for 30 min at 37 °C, then treated with 100 nM C10-NBD for another 30 min at 37 °C. In both cases, samples were harvested in cell stripper, washed once and resuspended in FACS buffer (PBS + 2%FBS). C10-NBD fluorescence was measured using BD FACSCalibur Flow Cytometry System. Results were analyzed using FlowJo_v10.7.1 and summarized as mean intensity ± SD. The variance across groups compared for statistical significance by Student’s *t* test.

### Confocal microscopy time-lapse or Z-stack

The Zeiss LSM 880 with airyscan confocal laser scanning microscope was used with a plan-apochromat ×40 (NA 1.3)/×63 (NA1.4) oil objective. Optimal sampling conditions for airyscan acquisition were achieved by selecting the super-resolution scanning modality [32]. The detailed methodology can be found in (Additional file 1).

### Protein quantification

Cells were plated in a 6-well or 10-cm dish. Treatment was added at 60% confluency for the allotted time. Then cells were harvested and lysed in lysis buffer (RIPA + protease inhibitor + 150 mM NaCl (total 300 mM). Protein concentrations were determined by BCA protein assay. Then, 4.5 μg of protein were loaded per well. Primary anti-SREBP2 and anti-LDLR antibodies were used at 1:15 dilution. Corresponding anti-rabbit or anti-mouse secondary antibodies were added. Densitometries from simple WES were normalized to total protein.

### RNA sequencing

Bulk RNA sequencing (RNAseq) was performed by the Genome Technology Access Center at the Washington University School of Medicine in St. Louis. Total RNA was isolated using TRIzol. Total RNA integrity was determined using Agilent Bioanalyzer or 4200 Tapestation. Library preparation was performed with 10 ng of total RNA with a Bioanalyzer RIN score greater than 8.0. ds-cDNA was prepared using the SMARTer Ultra Low RNA kit for Illumina Sequencing (Takara-Clontech) per manufacturer’s protocol. cDNA was fragmented using a Covaris E220 sonicator using peak incident power 18, duty factor 20%, cycles per burst 50 for 120 s. cDNA was blunt ended, had an A base added to the 3’ ends, and then had Illumina sequencing adapters ligated to the ends. Ligated fragments were then amplified for 12–15 cycles using primers incorporating unique dual index tags. Fragments were sequenced on an Illumina NovaSeq-6000 using paired end reads extending 150 bases. Features containing fewer than 10 reads were not included in the analysis. The RNAseq data generated in this study have been deposited in NCBI’s Gene Expression Omnibus and are accessible through GEO Series accession number GSE260557. Unbiased Gene Set Enrichment Analysis (GSEA) [33] were conducted against the Hallmark Gene Sets (MSigDB 2023.2.Hs), and the curated C5 gene ontology biological process (MSigDB C5.go.bp.v2023.2.Hs) made available by the Molecular Signatures Database (MSigDB). Normalized enrichment score and FDR q values generated by these analyses were visualized using heatmap created with GraphPad, enrichment plots generated by GSEA software (v4.3.3), and a pathway enrichment map created with Cytoscape using its EnrichmentMap, AutoAnnotate applications.

### Quantitative PCR

Cells were directly lysed in Trizol (Thermo Fisher Scientific) by pipetting up and down, while tumor tissue samples were homogenized in Trizol using Tissue Lyzer II (Qiagen). Total RNA was extracted according to the manufacturer’s instructions and was quantified using a Nanodrop spectrophotometer (Thermo Fisher Scientific). RNA was converted into cDNA using QuantiTech Reverse Transcription Kit (Qiagen). Quantitative real-time PCR (qRT-PCR) was performed on the CFX ConnectTM Real-Time PCR Detection System (Bio-Rad) using IQ SYBR Green Supermix and the primers listed in Table S1. The reaction protocol includes an initial preincubation at 95 °C for 3 min to denature the DNA, with amplification performed for 40 cycles (10 s at 95 °C, 10 s at 55 °C and 30 s at 72 °C). Gene expression levels were expressed as normalized values relative to the housekeeping gene *Rplo*. The raw dataset is summarized in an excel file (Additional file 8).

### Animal studies

Mouse protocols were approved by Washington University in St. Louis Institutional Animal Care and Use Committee (IACUC). Mice were maintained under IACUC guidelines. A syngeneic mouse model was used: 1.5 × 10^6^ KP2 cells were collected in a 1:1 DMEM:Matrigel solution then grafted by subcutaneous injection into the right flank of C56BL6 (female, 4–6 weeks old, injected at 5 weeks old, ~20 g). The treatment vehicle was 25% Kolliphor EL + 75% molecular grade water. Investigators were not blind to treatment groups. Length and width of resulting tumors were measured daily, and tumor volumes were calculated using the formula (0.5 × Length × Width^2^).

To investigate the effect of the combination of C10 and simvastatin on tumor size, cell line-derived allografts were allowed to grow to an average volume of 100 mm^3^, at which point, mice were randomly assigned to treatment groups of either vehicle daily, 60 mg/kg C10 intraperitoneal (IP) daily, 60 mg/kg simvastatin IP daily, or a combination of both C10 and simvastatin. Only tumors that reached the target volume within 14 days of injection were included. Tumors were allowed to grow to volumes of 2000 mm^3^ or until 21 days had passed. Simple randomization was achieved using an online tool (https://www.graphpad.com/quickcalcs/randomize2/) to generate a list randomized list that assigns each mouse to a group. Ten mice per treatment group were utilized for all studies. Sample size was calculated a priori using online sample size calculator (https://clincalc.com/stats/samplesize.aspx). To calculate differences between the means of two independent groups, as small as 20% change in mean (with SD = 15, alpha = 0.05; power = 0.80), sample size must be at least 9 per group. To account for tumors not growing or those that ulcerate early, *n* of 10 was decided a priori. Tumor growth curves are shown as mean ± SEM, with variance across groups compared for statistical significance by two-way ANOVA.

### Lipidomics analysis

Liquid chromatography-tandem mass spectrometry (LC-MS/MS) analytical techniques were used to measure cholesterol and cholesterol esters. Samples were submitted on dry ice to the Washington University in St Louis Division of Endocrinology, Metabolism, and Lipid Research. The analysis was performed on a Shimadzu 10Avp HPLC system and a SIL-20AC autosampler coupled to TSQ Quantum Ultra mass spectrometer (Thermo) operated in positive selected reaction monitoring (MRM) mode. Data processing was conducted with Xcalibur (Thermo). Lipids were extracted in the presence of appropriate internal standards: d7-cholesterol, and d7-CE (18:2) for cholesterol, CE, respectively. The sample aliquots for cholesterol were derivatized with nicotinic acid to improve mass spectrometric sensitivity. Quality control (QC) samples were prepared by pooling an aliquot of each study sample and injected every 5 study samples to monitor the instrument performance. Only the lipid species with %CV < 15% in QC injections are reported. The relative quantification data for all analytes were presented as the peak area ratios of each analyte to its internal standard.

For the in vitro samples, cell pellets (1 × 10^6^) were prepared from AsPC1, MiaPaCa, HPAC, KP2, OVCAR8, and SYO1 cells treated with 0, 5, 10, or 20 µM of C10 for 24 h. For the in vivo analysis, C57BL6 mice with KP2 tumors greater than 200 mm^3^ were randomly assigned to receive IP injections of either vehicle or C10 (60 mg/kg) in vehicle once daily for 3 days. The collected tumors (100–200 mg; quadruplicates) were homogenized in water (4 ml/g tumor) and the Blyth-Dyer extraction method was used to extract cholesterol, and CE from 50 µl of homogenate. The raw dataset is summarized in an excel file (Additional file 2). Quantification of analyses are summarized as mean ± SD, with variance across groups compared for statistical significance by Student’s *t* test.

### Statistical analysis

Statistical details of experiments can be found in the methods details for each experiment. Briefly, all experiments were conducted in triplicates with data presented as mean ± SD unless otherwise indicated. Variance across groups compared for statistical significance by Student’s two-tailed *t*-test for two groups, or one-way ANOVA for three or more groups, or two-way ANOVA when studying the effect of two factors on the outcome. *p* values less than 0.05 were considered statistically significant. The symbols ns (not significant), *, **, ***, and **** represent *p* values >0.05, ≤0.05, ≤0.01, ≤0.001, and ≤0.0001 respectively.

## Results

### S2L cytotoxicity across cancer types does not correlate with S2R affinity

**Fig. 1:**
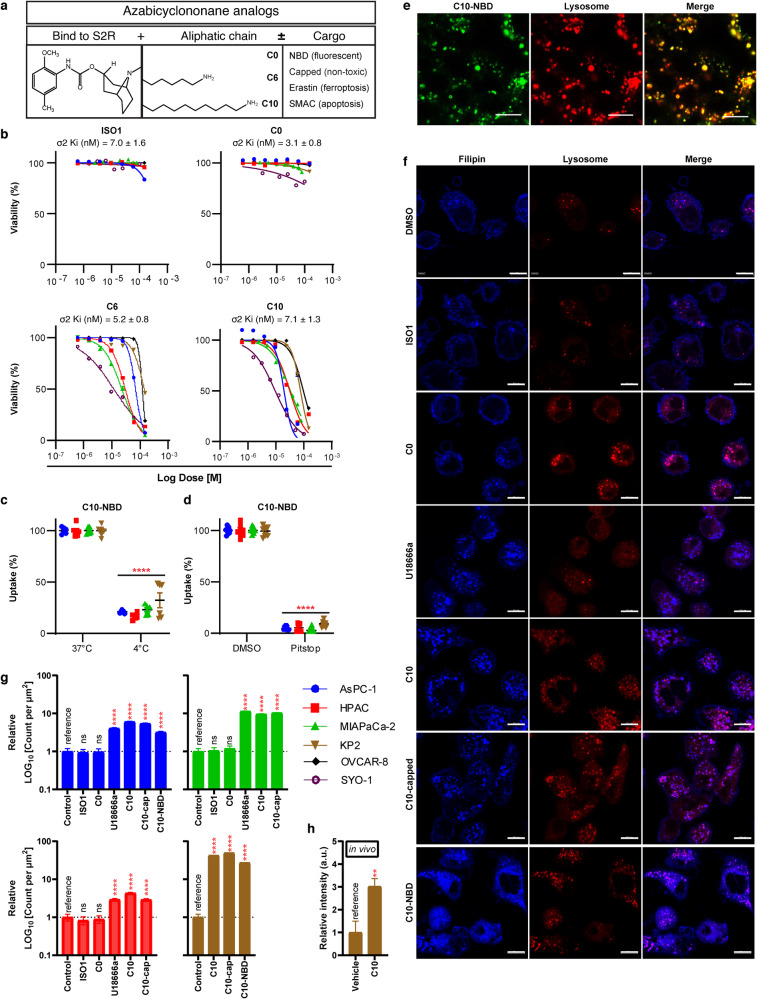
Characterization of S2L cytotoxicity, uptake, and effect on free cholesterol across multiple cancers. **a** Schematic diagram depicting the structure and functions of different components of the azabicyclononane class of S2L. **b** Proliferation curves of pancreatic (AsPC-1, HPAC, MIAPaCa-2, KP2), ovarian (OVCAR-8), and synovial sarcoma (SYO-1) cell lines treated with various S2L for 24 h. Data represents mean ± SD; *n* = 3. Flow cytometry measuring C10-NBD [100 nM, 30 min] fluorescence after incubation at 4 °C (*n* = 9) (**c**) or pretreatment with pitstop-2 (*n* = 6) (**d**). Data represent mean ± SD. **e** Representative confocal microscopy images from HPAC cells treated with 100 nM C10-NBD for 20 min counterstained for lysosome. Scale bar equals 20 µm. **f** Representative confocal microscopy images from AsPC-1 cells treated with 10 µM various compounds for 24 h, stained with filipin and counterstained for lysosome. Scale bar equals 10 µm. Bar graph quantifying count of filipin puncta per µm^2^ in AsPC-1 (seen in **f**), HPAC, MiaPaCa-2, and KP2 in a cellular model treated with 10 µM of S2L for 24 h (**g**) and a mouse model C57BL6/KP2 treated with 60 mg/kg C10 for 21 days (**h**). Data represent mean ± SD (*n* = 10 cells). ns not significant; **p < 0.01; ****p ≤ 0.0001.

Sigma-2 receptors (S2R) are overexpressed across cancer types [2]; however, the broad structural diversity among the S2 ligands have been reported to cause different biologic effects [34], despite having equivalent affinities to the S2R [35]. To corroborate these findings, the cytotoxicity of four S2L was tested in a panel of cancer cell lines, including pancreatic cancer (AsPC-1, MIAPaCa-2-2, HPAC_human; KP2_mouse), ovarian (OVCAR-8_human), and synovial sarcoma (SYO-1_human). The ligands studied were a benzamide analog, ISO1, used primarily for tumor imaging purposes [4], and multiple azabicyclononane analogs, which all share the same azabicyclononane moiety responsible for binding to the S2R, but have distinct lengths of aliphatic side chains C0, C6, and C10. The latter two S2L backbones could be further conjugated to different drug cargoes to form distinct S2L-conjugates (Fig. 1a, see Additional file 3 for structural information and nomenclature definitions).

Despite having similar high binding affinities to the S2R ranging between 3.1–7.0 nM (Fig. 1b), these ligands exhibited distinct bioactivities across the various cancer types. Both ISO1 and C0 did not show signs of cytotoxicity even at doses as high as 150 µM neither at 24 h (Fig. 1b), nor at 72 h (Additional file 4). In contrast, C6 and C10 caused dose-dependent cell death (Fig. 1b) in accordance with our previously published work [8, 36]. It is noteworthy, that within the azabicyclononane class of compounds, the biologic activity was directly correlated with the length of the aliphatic side chain: C10 > C6 >>> C0. (e.g., AsPC-1 24 h IC50 C10 19.6 µM vs. C6 68.9 µM vs. C0 > 150 µM). Moreover, while S2L conjugates with toxic cargoes (C6-E, C10-SMAC) induced potent killing across all the cell lines, conjugates of C10, whether with fluorescent cargo (C10-NBD) or an inert non-toxic cargo (C10-capped) were less potent than C10 alone and only showed killing after 72 h of killing (Additional file 4).

Since our laboratory is focused on utilizing S2L for cancer-targeted drug delivery, the fluorescently tagged C10 variant, C10-NBD, was primarily employed for visualizing S2L uptake and subcellular localization. C10-NBD was quickly taken up by the cancer cells but was significantly inhibited at 4 °C (Fig. 1c), suggestive of an energy-dependent uptake mechanism. Similarly, C10-NBD uptake was almost completely inhibited (~95%) in the presence of the endocytosis inhibitor pitstop-2 (Fig. 1d). Lastly, confocal microscopy revealed that, within 20 min of treatment, C10-NBD rapidly localized to the lysosomes when monitored via live-cell imaging (Fig. 1e); however, C10-NBD did not localize to the nucleus, mitochondria, nor the endoplasmic reticulum within that timeframe (Additional file 5). These data indicate that S2L are internalized via energy-dependent, receptor-mediated endocytosis, ultimately reaching the lysosomal compartment of the cells.

### C10 induces an increase in intralysosomal free cholesterol

Recently, a cholesterol derivative (20(S)-hydroxycholesterol) was identified as an endogenous ligand of S2R/TMEM97 [37], which in turn was reported to be involved in cholesterol homeostasis [14]. Because of the uptake pattern of S2L into the lysosomal compartment (Fig. 1e) and its association with the S2R/TMEM97 which physically interacts with LDLR [16] and NPC1 [15], which in turn, internalizes cholesterol to the lysosome and shuttles free cholesterol out of the lysosome, respectively, we hypothesized that cytotoxic S2L may be affecting cholesterol homeostasis and asked whether S2L exposure might be disrupting intracellular cholesterol trafficking and/or processing.

To this end, the effect of S2L treatment on the intracellular distribution of free cholesterol was visualized using filipin stain [38]. Confocal microscopy revealed that in the DMSO control group, free cholesterol was found mainly lining the plasma membrane with minimal amounts found intracellularly, a staining pattern seen also with non-cytotoxic S2Ls ISO1 and C0 (Fig. 1f). In contrast, C10 significantly increased the intracellular filipin signals, which happened to co-localize with a lysosomal marker (Fig. 1f). This finding could be interpreted as either a C10-induced increase in LDL internalization via LDLR, or a C10-induced blockade of cholesterol egress from the lysosome; the latter resembling the effects of NPC1 inhibition with U18666a [23]. Indeed, U18666a shared a similar phenotype as C10 (Fig. 1f). Interestingly, both C10-NBD and C10-capped also induced an increase in intralysosomal free cholesterol (Fig. 1f). Quantifying the number of intracellular filipin puncta confirmed the significant increase in free cholesterol induced by U18666a, C10, and the C10 conjugates (Fig. 1g). Moreover, there was a significant 3-fold increase in filipin signal in an in vivo model of C57BL6/KP2 treated with 60 mg/kg C10 for 21 days (Fig. 1h).

As the non-cytotoxic ligands (ISO1, C0) failed to induce changes in cholesterol distribution, this suggests that this phenotype is most likely (1) involved in the C10-induced cytotoxicity, (2) the result of the aliphatic side chain, and (3) chemically linking various cargo to the side chain might not alter its ability to induce this cholesterol phenotype. Nonetheless, the mechanism of this increase in intralysosomal cholesterol is yet to be determined.

### C10 enhances uptake of cholesterol rather than blocking its trafficking from the lysosome

LDLs rich in cholesterol esters are internalized into cells via LDLR-mediated endocytosis and are hydrolyzed in the lysosomes upon which the resultant free cholesterol is shuttled via NPC1 to the ER where the major sensor regulator of cholesterol homeostasis resides (SREBP2-SCAP-INSIG complex) [22]. Any excess of free cholesterol at the ER is esterified by ACAT1 to cholesterol esters that are either stored in lipid droplets across the cytoplasm or released into the extracellular milieu in association with lipoproteins [22]. However, pharmacologic inhibition of NPC1 prevents cholesterol esterification due to the obstruction of free cholesterol transfer from the lysosome to the ER [23]. Therefore, since both C10 and the NPC1 inhibitor U18666a shared similar filipin staining phenotypes (Fig. 1f, g) and were both cytotoxic, albeit C10 being slightly more potent than the latter (Additional file 6), we sought to investigate the putative mechanistic differences between the two compounds by quantifying the levels of free cholesterol and cholesterol esters.

**Fig. 2:**
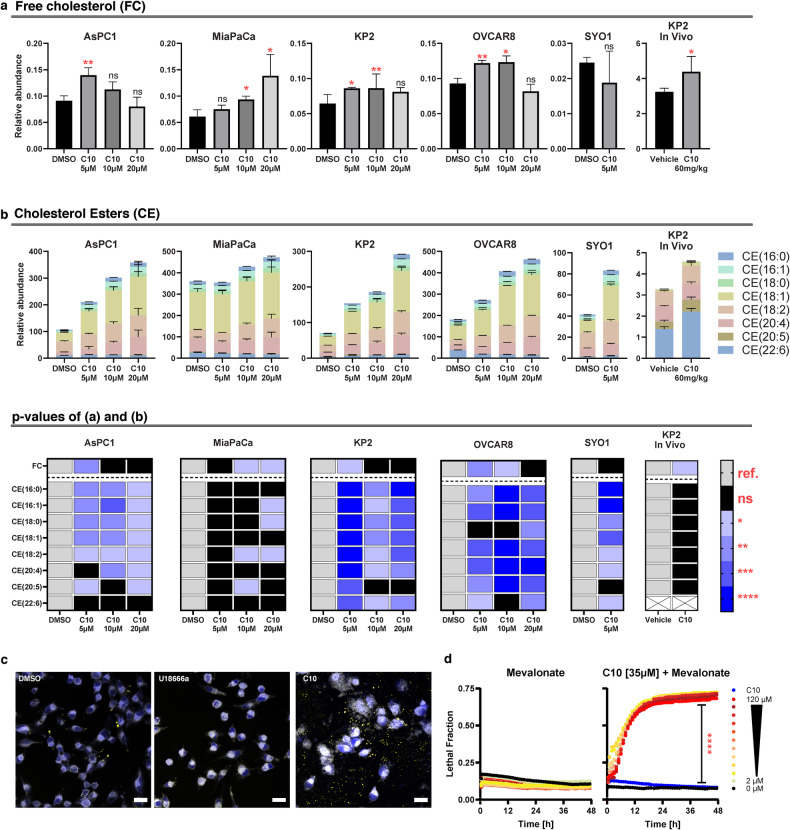
Lipidomics analysis identifies changes in free cholesterol and cholesterol esters upon C10 treatment. Bar graphs showing levels of free cholesterol (**a**) and cholesterol esters (**b**) in cells treated with the indicated dose of C10 for 24 h (*n* = 3), or KP2 tumor samples derived from C57BL6 mice after treatment with C10 [60 mg/kg] for 3 days via intraperitoneal injection (*n* = 4). Data are presented as means ± SD. Heatmap summarizing the statistical analysis of (**a**, **b**). The white boxes marked with X indicate that the metabolite was not analyzed (i.e., CE(22:6) in the in vivo model). **c** Representative confocal microscopy images from MiaPaCa-2 treated with DMSO, U18666a, or C10 for 24 h, showing the merged image of blue and [yellow] channels from filipin and [Nile Red], measuring free cholesterol and [cholesterol esters] respectively. Scale bar equals 20 µm. **d** Cell death over time of AsPC-1 cells treated with a serial dilution of mevalonate (DF = 1.5) alone [left] or in combination with a sublethal dose of C10 [right]. Data represent mean ± SD; *n* = 3. ref. reference, ns not significant; *p < 0.05; **p < 0.01; ***p ≤ 0.001; ****p ≤ 0.0001.

Lipidomics analysis revealed that C10 induced a significant increase in free cholesterol levels across cancer cell lines that was no longer significant at higher doses (Fig. 2a). This decline in free cholesterol levels was parallelled by a significant increase in levels of cholesterol esters in a dose-dependent manner (Fig. 2b), suggestive of unhindered cholesterol trafficking and esterification processes after treatment with C10. To corroborate these findings in vivo, C57BL6 mice bearing KP2 pancreatic flank tumors were treated for 3 days with C10. Lipidomics analysis of those tumors revealed a significant increase in free cholesterol (*p* = 0.0452). While the cholesterol ester levels were trending upward, their increase did not reach statistical significance (Fig. 2, See Additional file 2 for the full dataset). Additionally, a complementary immunohistochemical assay was utilized to visualize the distinct effects of C10 vs. U18666a on the levels of free cholesterol and cholesterol esters with filipin and Nile Red stains respectively. As demonstrated earlier, both C10 and U18666a had a significant increase in free cholesterol (Fig. 1f, g). Conversely, qualitative observations of Nile Red stains revealed that U18666a reduced the baseline levels of cholesterol esters to a similar or even slightly lower level than the untreated control; however, the amount of cholesterol esters detected in C10 treated samples was significantly higher than the controls (Fig. 2c, Additional file 7). These findings further suggest that the mechanism of action of C10 appears to be fundamentally different than that of an NPC1 inhibitor.

Since both C10 and U18666a induce cancer cytotoxicity by disrupting cholesterol homeostasis possibly through distinct mechanisms, we sought to further differentiate these two compounds by treating cancer cells with C10 in combination with mevalonate, especially since (1) mevalonate is the product of the rate limiting step catalyzed by HMGCR in the de novo synthesis pathway of cholesterol, and (2) mevalonate supplementation was previously reported to provide significant neuroprotection to cells treated with U18666a. Therefore, if the C10-dependent cytotoxicity was the result of low-cholesterol status resulting from blockade of intracellular cholesterol trafficking (similar to the NPC1 inhibitor), then supplementing the media with mevalonate was expected to protect from cell death. However, the combination of C10 with increasing doses of mevalonate resulted in significant cell death augmentation, which was similar in the combination with the lowest dose (2 µM) and the highest dose (120 µM) of mevalonate (Fig. 2d).

Together, these findings strongly suggest that, in stark contrast to NPC1 inhibition with U18666a, C10 has unimpeded intracellular cholesterol trafficking, as evidenced by the presence of cholesterol esters on both lipidomics and immunohistochemistry analyses, and C10-induced cytotoxicity is most likely mediated by an excess of intracellular free cholesterol.

### Transcriptional profiling identifies S2L-induced alterations in ER stress and cholesterol homeostasis

**Fig. 3:**
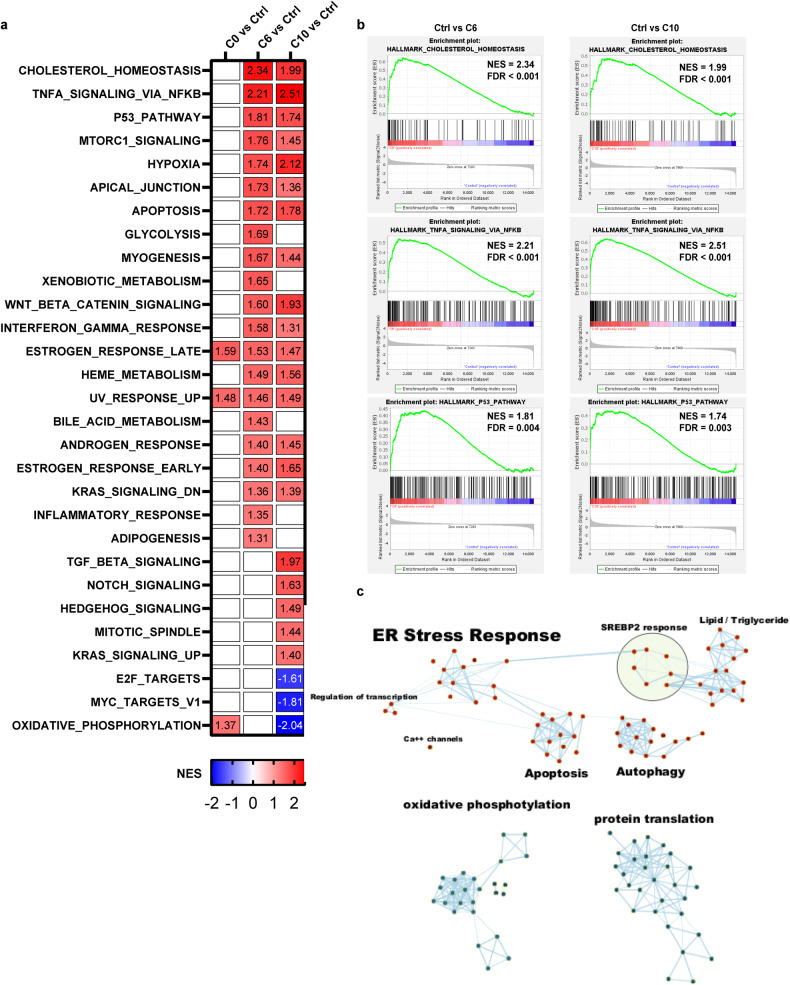
Gene set enrichment analysis of transcriptional profiles discriminates between cytotoxic and non-cytotoxic S2L. **a** Heatmap of normalized enrichment scores (NES) for the 50 Hallmark pathways in SYO-1 cells treated with 10 µM of C0, C6, or C10 for a 2-h time interval. Pathways that were significantly enriched (*p* < 0.05, FDR < 0.25) were colored either in red for upregulated pathways or blue for downregulated pathways in the S2L relative to the control (DMSO). **b** Gene set enrichment plots of the top three highly significant hallmark gene sets that were upregulated in both C6 and C10. **c** Enrichment map of the functional analysis of the gene ontology biologic process (GOBP) displaying the enriched gene-sets in C10-treated vs. Control. Pathways are shown as nodes that are colored according to enrichment scores: red represents enrichment in C10-treated cells (i.e., up-regulation after C10 treatment), whereas blue represents enrichment in control cells (i.e., down-regulation after C10 treatment). Edges represent shared genes between the connected pathways. Functionally related gene-sets were clustered and assigned a label.

To investigate the mechanism of S2L-mediated cytotoxicity in an unbiased fashion, RNA sequencing technology (RNAseq) was employed to establish any differences in the transcriptional landscape between cancer cells treated with either the non-cytotoxic C0, or the cytotoxic C6 and the more potent C10. Following a short-term (2-h long) drug exposure, the cells were harvested and processed for transcriptome and functional gene enrichment analysis. Gene set enrichment analysis (GSEA) against Hallmark gene ontology sets implicated the activation of cholesterol homeostasis, TNFa signaling via NFkB, and P53 signaling pathways in response to both C6 and C10 treatment (Fig. 3a, b; *p* < 0.05 and FDR < 0.005).

To determine the signature molecular pathways that are predominantly involved in the cytotoxicity of C10, GSEA was performed with a focus on gene ontology biologic processes (GOBP). Of the 7643 gene sets evaluated, 5608 were upregulated (679 of those with *p* < 0.05 and FDR < 0.25); 2026 were downregulated (48 of those with *p* < 0.05 and FDR < 0.25). As these enriched pathways are inherently redundant, EnrichmentMap in Cytoscape was used to group similar pathways that share the same genes into major biological clusters. The enrichment map shows 5 groups of overexpressed pathways: ER stress response, SREBP2 response, apoptosis, autophagy, and transcription regulation; and 2 groups of underexpressed pathways: protein translation, and oxidative phosphorylation (Fig. 3c, see Additional file 8 for a detailed description of Hallmark and GOBP analyses). Therefore, this functional analysis suggested that ER stress response is the central biological effect of C10-induced cytotoxicity.

### C10 causes activation of ER stress markers

**Fig. 4:**
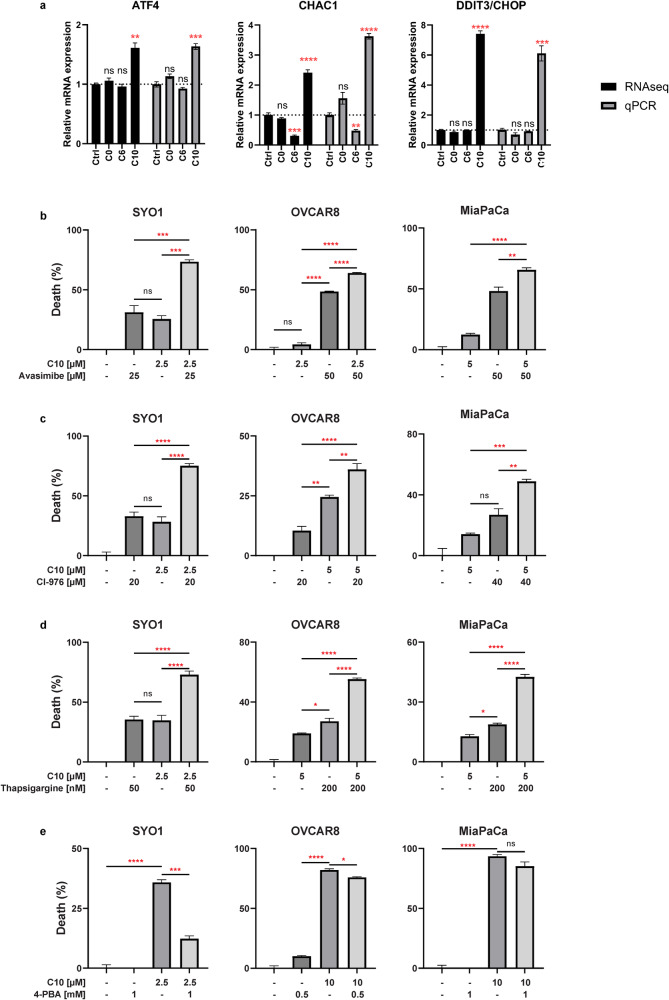
C10 induces ER stress response. **a** Differentially expressed mRNA from RNAseq (black) or qPCR (gray) of SYO1 cells treated with the different S2L. Data are presented as mean ± SD; *n* = 3. Percent of cell death from Cell TiterGlo assay measuring the viability of SYO1, OVCAR8, and MiaPaCa-2 cells pretreated with avasimibe (**b**), CI-976 (**c**), thapsigargin (**d**), or 4-PBA (**e**) for 2 h then treated with C10 for 24 h. Data represent mean ± SD; *n* = 3. ns = *p* > 0.05; *p ≤ 0.05; **p ≤ 0.01; ***p ≤ 0.001; ****p ≤ 0.0001.

Transcriptional profiling suggested that C10 seemed to be involved in triggering ER stress via upregulation of ATF4, CHAC1, and DDIT3/CHOP (Fig. 3). First, these markers were validated using q-PCR. C10 was found to induce a robust increase in mRNA expression levels of all three markers of ER stress (Fig. 4a). Next, these new findings were interrogated with a multi-pronged approach. Cell viability assays were employed to investigate the effect of ER stress modulators (inducers or inhibitors) on the baseline cytotoxicity of C10. It was anticipated that if single agent treatment with C10 was activating ER stress, then adding an ER stress inducer would further enhance its cytotoxicity profile, while adding an ER stress inhibitor would ameliorate S2L bioactivity and protect from cell death induction.

First, C10 was combined with ACAT inhibitors, which induce ER stress by blocking cholesterol esterification and leading to the accumulation of free cholesterol in the ER [39]. Cell viability assays of C10 in combination with avasimibe, a dual ACAT1/2 inhibitor, resulted in augmentation of cell death in all three cell lines tested (Fig. 4b). A similar result was obtained with CI-976, a selective ACAT1 inhibitor, which also significantly increased cell death of C10 (Fig. 4c). Furthermore, when C10 was combined with thapsigargin, an ER stress inducer that inhibits the Sarco-Endoplasmic Reticulum Calcium ATPase (SERCA) [40], a significant augmentation of cell death was accomplished (Fig. 4d).

By employing an inverse approach, a chemical chaperon, 4-phenylbutyric acid (4-PBA), which can reduce ER stress by enhancing the protein folding process [41], was utilized to counteract the baseline cytotoxicity of S2L (C10). A cell viability assay confirmed that 4-PBA could indeed reduce the cytotoxicity of C10 in two out of three cancer cell lines (Fig. 4e). These data suggest that C10 does indeed induce an ER stress response; however, the exact mechanism of this activation process has yet to be determined.

### C10-induced cholesterol excess mediates ER stress that activates SREBP2 as an adaptive response against cell death

**Fig. 5:**
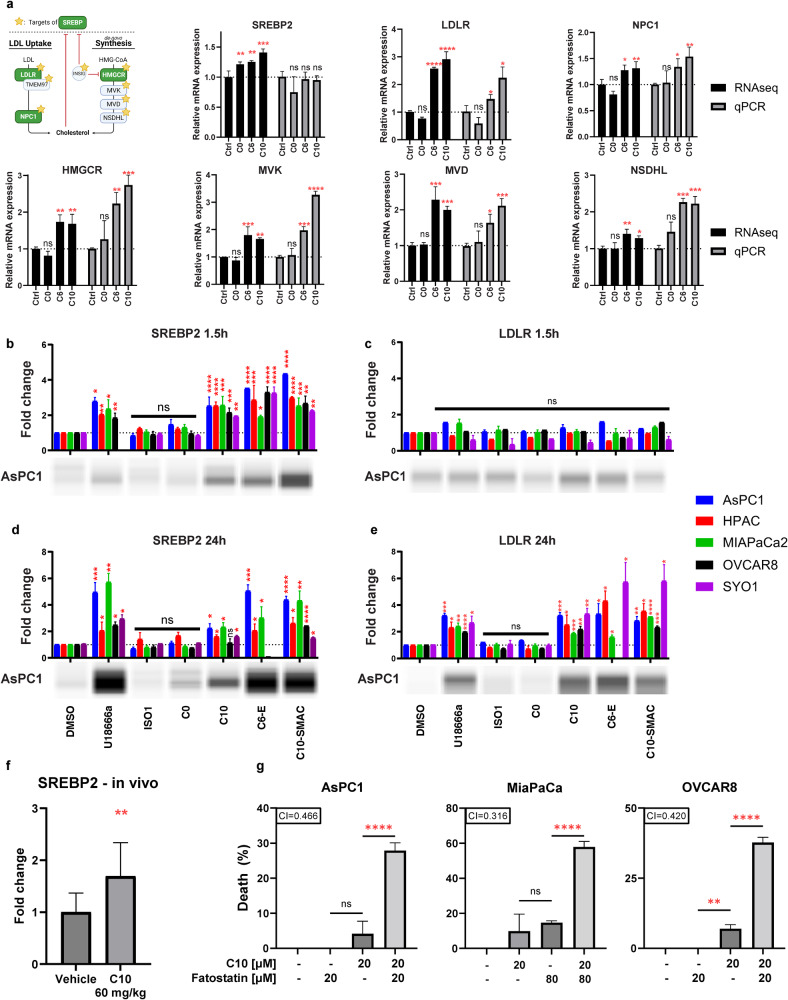
C10 induces SREBP2 activation as an adaptive response downstream of ER stress response. **a** Differentially expressed mRNA from RNAseq (black) or qPCR (gray) of SYO1 cells treated with the different S2L. Data are presented as mean ± SD; *n* = 3. **b**–**e** Bar graph of protein expression of cleaved SREBP2 and mature LDLR across multiple cell lines treated with 10 µM of different S2L for 1.5 h or 24 h. Protein levels were determined via densitometry then normalized to the total protein amount per sample. A representative blot from AsPC-1 is displayed below its respective bar graph. Data represent mean ± SD; *n* = 3. **f** Bar graph of protein expression of cleaved SREBP2 in KP2 tumor samples derived from C57BL6 mice after treatment with C10 [60 mg/kg] for 21 days via intraperitoneal injection (*n* = 12; two statistical outliers were eliminated from the control using ROUT method with *Q* = 1%). **g** Percent of cell death from Cell TiterGlo assay measuring the viability of AsPC-1, MiaPaCa-2 and OVCAR-8 cells after 24 h treatment with C10 ± 2 h pretreatment with Fatostatin. The combination index (CI) is displayed with the respective bar graph. CI values less than, equal to, or greater than 1 ± 0.1 indicate synergy, additivity, or antagonism, respectively. Data represent means ± SD; *n* = 9. ns = *p* > 0.05; *p ≤ 0.05; **p ≤ 0.01; ***p ≤ 0.001; ****p ≤ 0.0001.

Since cytotoxic S2L were found to upregulate cholesterol homeostasis pathways (Fig. 3a, b), and cholesterol excess could lead to activation of ER stress response [24], we hypothesized that C10 may be inducing ER stress by disrupting cholesterol homeostasis. First, the SREBP2 transcription factor, and six of its downstream target genes were selected for qPCR validation. While C6 and C10 treatment did not cause a significant change in SREBP transcription factor expression at the qPCR level, there was a significant increase of multiple of its downstream targets involved in cholesterol uptake (LDLR, NPC1) and cholesterol synthesis (HMGCR, MVK, MVD, NSDHL) (Fig. 5a), signifying that C10 might induce the activation of the available SREBP2 proteins rather than inducing gene transcription.

To verify this hypothesis, Western blotting was used to measure the protein levels of cleaved/activated SREBP2 and LDLR after 1.5 and 24 h treatment with various S2L and NPC1 inhibitor (as a control), which was predicted to activate SREBP2 and subsequently LDLR due to a low-cholesterol status sensed at the ER [23]. As expected, cells treated with U18666a responded with a quick increase in SREBP2 levels (1.5 h) and a subsequent increase of LDLR (24 h), confirming that blocking cholesterol from reaching the ER can cause SREBP2 activation and upregulation of its downstream targets (Fig. 5b–e). Similarly, upon treatment with C10, there was a significant 2.0–2.5 fold increase in SREBP2 activation within 1.5 h of treatment and 2.5–5.7 fold increase at 24 h (Fig. 5b, d). This increase was also seen in an in vivo model of C57BL6/KP2 treated with 60 mg/kg C10 for 21 days (Fig. 5f). Similarly, when tested in OVCAR8 and SYO1 cells, C6 induced a significant increase in SREBP2 and LDLR (Additional file 9).

Conversely, there was no significant difference in mature LDLR protein levels (mLDLR) at the early timepoint (Fig. 5c); however, C10 induced a significant (>2.0-fold) increase in mLDLR after 24 h post treatment (Fig. 5e). The most likely scenario for this finding is a delay from LDLR mRNA translation into LDLR monomers (50 kDa), which subsequently combine into the premature form LDLR (120 kDa), followed by glycosylation events that are required to generate the fully functional and mature form of LDLR (mLDLR, 160 kDa) (see Additional file 9 for the full and uncropped Western blots showing SREBP2 (cleaved and uncleaved) as well as LDLR (monomers, premature, and mature).

Interestingly, upon treatment with non-cytotoxic ISO1 or C0, there was no significant difference in the levels of neither the cleaved SREBP2 nor mLDLR at both timepoints and across multiple cancer cell lines tested (Fig. 5b–e), suggesting that activation of the cholesterol pathway was unique to the cytotoxic effects of S2Ls. Of note, the S2L conjugates C6-Erastin, C10-SMAC, C10-capped, and C10-NBD also induced significant increases in SREBP2 and mLDLR protein levels (Fig. 5b, d, e, Additional file 9). This latter finding is highly remarkable, as it suggests that the S2L delivery moieties of the respective drug conjugates resemble the isolated ligands when it comes to their biological consequences, even after their conjugation with a respective drug cargo.

Next, the underlying mechanism of the SREBP2 activation induced by the cytotoxic S2L was further investigated. It is widely accepted that SREBP2 is activated due to low-cholesterol levels at the ER, including with NPC1 dysfunction, to enhance the uptake and synthesis of cholesterol [22]. It has also been reported that the ER stress response [42], which can be activated by an excess of free cholesterol at the ER, can induce the activation of SREBP2 via three suggested mechanisms (reviewed elsewhere) [42, 43] despite the initial cholesterol excess. This can act as a cytoprotective adaptive response, since the resulting increase in phospholipids is needed to expand the ER membrane and restore protein folding [42, 43]. To investigate the hypothesis that SREBP2 activation was downstream of the ER stress response, SREBP2 was pharmacologically inhibited with fatostatin [44]. If SREBP2 activation was a compensatory mechanism, fatostatin was expected to enhance the cytotoxicity of C10. When pancreatic and ovarian cancer cells were treated with fatostatin in the presence of C10, a significant synergistic cell death was noted (Fig. 5g). This suggests that SREBP2 was activated by ER stress as an adaptive compensatory mechanism.

To support these new discoveries, the kinetics of the increase in cholesterol levels were compared to the dynamic changes in LDLR protein levels. Filipin stain of AsPC1 cell treated with C10 at multiple timepoints revealed a rapid increase in free cholesterol that peaked around 2 h and then started to decline over time (Additional file 10). This decrease was most likely due to the unaltered cholesterol esterification process following C10 exposure (Fig. 2). More importantly, the early (2 h) increase in cholesterol levels was occurring prior to the increase in LDLR protein levels (Fig. 5c). Therefore, we conclude that the C10-induced acute increase in cholesterol levels is not mediated through the SREBP2/LDLR signaling axis, but rather due to an increased uptake kinetics of LDL via the already available mLDLR. This corroborates the findings of Riad et al., who have previously demonstrated that the rate of LDL uptake by LDLR is significantly increased through the formation of the trimeric S2R/TMEM97-PGRMC1-LDLR complex [16].

Taken together, these findings suggest that the initial event in C10-based cytotoxicity is an increase in free cholesterol, which is transported normally to the ER, where its excess leads to the activation of the ER stress response, which in turn activates SREBP2 as an adaptive measure to counteract cell death.

### Abrogating the ER-stress-induced adaptive response (SREBP2) potentiates C10 cytotoxicity

**Fig. 6:**
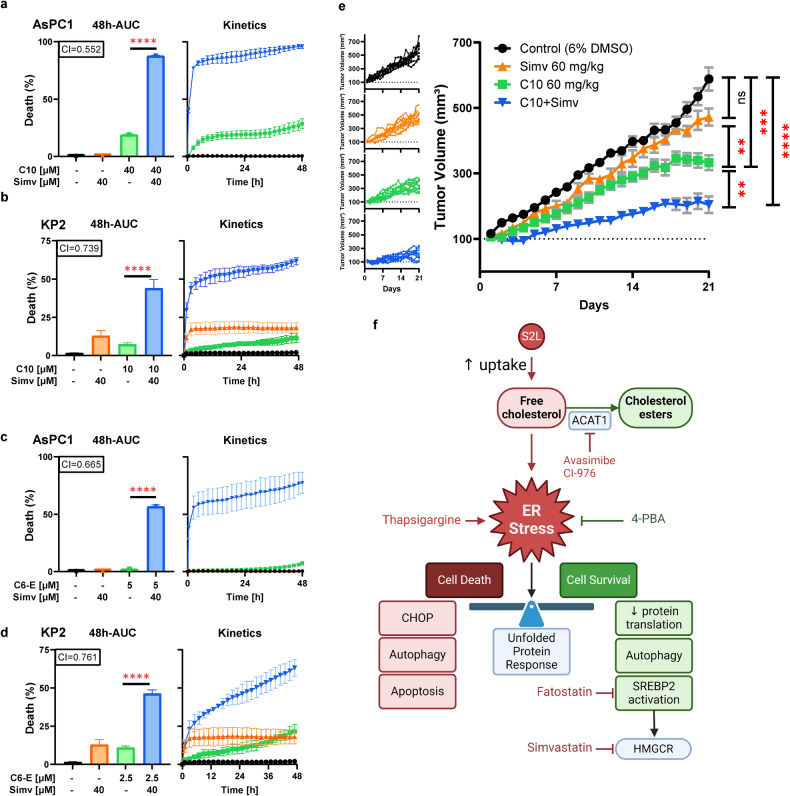
Abrogating the ER-stress-induced adaptive response (SREBP2) potentiates C10 cytotoxicity. Percent of cell death from the 48h-AUC [left] of lethal fraction over time [right] in AsPC-1 (**a**, **c**) or KP2 (**b**, **d**) cells treated with a S2L (C10 or C6-E), simvastatin, or a combination of both. The combination index (CI) is displayed with the respective bar graph. CI values less than, equal to, or greater than 1 ± 0.1 indicate synergy, additivity, or antagonism, respectively. Data represent mean ± SD; *n* = 3. **e** Tumor volume of KP2 xenografts (*n* = 10) under various conditions: vehicle, simvastatin, C10, or C10 combined with simvastatin. Data are represented by means ± SEM. **f** Schematic visualization of the study, prepared with BioRender. ns not significant (*p* > 0.05); **p ≤ 0.01; ***p ≤ 0.001, ****p ≤ 0.0001.

Since the S2L-induced SREBP2 activation and subsequent increase in cholesterol was found to be an adaptive response, we hypothesized that targeting the downstream targets of SREBP2 pharmacologically could result in drug combinations with potentially synergistic activity profiles as a means to enhance the therapeutic potency of S2L and, by extension that of S2L-based drug conjugates. Indeed, when sublethal doses of C10 were combined with simvastatin, the inhibitor of HMGCR [45], synergistic activity was achieved (Fig. 6a, b). Subsequently, we tested whether combining simvastatin with the drug conjugate C6-Erastin will result in a similar increase in cytotoxicity. Indeed, when AsPC1 cells were treated with a sublethal C6-Erastin dose in combination with low-dose simvastatin, synergistic cell death was documented (Fig. 6c, d). These results substantiate the new finding that the drug conjugates retain the ability of the parental sigma-2 ligands to cause similar cholesterol-induced ER stress.

Encouraged by these in vitro results, we investigated if combination therapy of C10 and simvastatin would lead to enhanced therapy outcomes in a syngeneic, immunocompetent in vivo model of murine PDAC using KP2 cells injected subcutaneously into C57BL6 mice. At clinically relevant simvastatin doses, there was no significant difference in tumor sizes between mice treated with simvastatin and the control group; however, C10 single-agent therapy led to a significant reduction in tumor growth (Fig. 6e). More importantly, treatment of mice with a combination of C10 and simvastatin resulted in the strongest therapy outcome among all groups, illustrated by a pronounced delay in tumor growth kinetics (Fig. 6e).

## Discussion

To date, there are more than 650 different S2L described in the literature [35]; yet the biologic effects of S2L can be quite diverse and cannot necessarily be predicted solely based on affinity data to the S2R. For example, while some S2L were found to induce proliferative, pro-survival, and anti-apoptotic effects on tumor cells [46], other S2L were found to induce tumor-selective cytotoxicity [47–51]. Furthermore, a subtle structural modification of S2L resulted in a functional conversion from a cytotoxic into a metabolically stimulating compound, highlighting the seemingly dual functional role of the sigma-2 receptor [34, 52]. Consequently, the primary goal of our current study was to elucidate reason(s) for the difference in cytotoxicity of structurally diverse S2L with similar affinities to the S2R (Fig. 1a, b). We found that cytotoxic S2L, such as C6 and C10, cause disturbances in cellular cholesterol homeostasis by inducing enhanced uptake of free cholesterol through the LDLR (Fig. 1f–h). Moreover, while S2L localized via energy-dependent receptor-mediated endocytosis to the lysosome (Fig. 1c, d) where the S2R/TMEM97 physically interacts with NPC1 protein [15], our study showed that C10 treatment did not appear to affect NPC1 function, and allowed for an uninterrupted intracellular cholesterol trafficking of free cholesterol to the ER, where it undergoes esterification by ACAT1 (Fig. 2). It is noteworthy that MiaPaCa cells had a significantly higher baseline level of cholesterol esters compared to all other cell lines (Fig. 2a, b), which corroborates the findings of Li et al., who showed that this particular cell line was characterized by substantially higher baseline levels of ACAT1 [39]. This resulted in MiaPaCa being the only cell line that exhibited a significant dose-response increase in free cholesterol levels, while having a less robust increase in cholesterol esters as the other cell lines, likely because the upregulated ACAT1 is already at maximum rate of conversion and the C10-induced increase in free cholesterol cannot be esterified as rapidly leading to its accumulation (Fig. 2a, b). Furthermore, the increased influx of cholesterol eventually results in overwhelming the esterification capabilities of the cell and the accumulating free cholesterol activates ER stress response (Fig. 3), which was found to be affected by ER stress modulators (Fig. 4). The activation of ER stress response is additional evidence that C10 is mechanistically distinct from NPC1 inhibitor as mouse and cellular models of Niemann-Pick type C disease are unable to activate the ER stress response [53]. Interestingly, the non-cytotoxic compounds (ISO1, C0) did not trigger such a phenotype (Figs. 1f, g, 3a, 4a and 5a–e), suggesting that this model of excess free cholesterol—ER-stress—SREBP2-activation is the mechanism of S2L cytotoxicity (Fig. 6f). It would be interesting to study if these findings are present in TMEM97 knockout cell lines especially since TMEM97-LDLR-PGRMC1 trimer is most likely responsible for rapid enhancement of cholesterol uptake leading to the increase in free cholesterol seen with C10 [16]; yet the genetic deletion of TMEM97 and PGRMC1 did not reduce the cytotoxicity of C10 [54].

The C10-induced SREBP2 activation and upregulation of its downstream pathway (Fig. 5a–f) was found to be an adaptive response (Fig. 5g), even though an excess of cholesterol is believed to cause an activation of ER stress. Essential pathways upregulated by SREBP2 lead to the accumulation of cholesterol, triglycerides, fatty acids, and phospholipids, suggested to be cytoprotective following cytotoxic stress [43]. For instance, the distal metabolites of cholesterol biosynthesis, including 7-dehydrocholesterol, can protect cells from phospholipid peroxidation and ferroptosis [55]. Additionally, the accumulation of fatty acids and lipid droplet biogenesis were found to enable cell survival during hypoxia [56]. Moreover, lipid droplets were reported as antioxidants that can protect triple-negative breast cancer cells against lipotoxic stress [57]. An additional target of SREBP2 is fatty acid synthase which induces de novo synthesis of lipids resulting in gemcitabine resistance in pancreatic cancer [58]. All of these protective mechanisms are downstream of SREBP2; therefore, inhibiting it directly with fatostatin (Fig. 5g), or inhibiting one of its target genes, HMGCR, with simvastatin (Fig. 6) was found to be synergistic when combined with C10. Additional research is needed to elucidate the exact mechanism behind the synergy of C10 with simvastatin.

S2L have been reported to induce cytotoxicity by multiple mechanisms, including lysosomal membrane permeabilization (LMP) [59], autophagy, and apoptosis [60]. S2L also stimulated a release of calcium from the endoplasmic reticulum [47]. Furthermore, C10 was reported to synergize with gemcitabine in pancreatic cancer [36]. Yet the exact molecular basis explaining this plethora of effects induced by S2L remained elusive. Interestingly, ER stress is also reported to induce LMP, sustained increase of cytoplasmic Ca^2+^, autophagy, and both caspase-dependent and independent apoptosis [25, 61]. Moreover, inducing ER stress by various methods, including thapsigargin or FASN inhibitors, resulted in synergy with gemcitabine [58]. For this reason, along with all the findings from our study, it can be concluded that the main mechanism of cytotoxicity of C10 (SW43) is induction of ER stress which might be the mechanism of synergy with gemcitabine in PDAC. This also supports the suggested hypothesis that S2R, like S1R, might be gatekeepers of ER stress [62] and corroborates the findings Li et al. that showed that another S2L, A011, induced ER stress in breast cancer [63].

As of today, experimental evidence from published work suggests that the superior potency of S2L drug conjugates was primarily mediated by the enhanced delivery kinetics/efficacy of the chemically linked effector molecules (cargoes) into the cancer cells [8, 10, 11]. However, the precise impact of the S2L moiety of C6-Erastin was not determined. It was remarkable to find that in two relevant drug conjugates, i.e., C10-SMAC and C6-Erastin, the latter currently being pursued for clinical development (ACXT-3102), the S2-moiety fully retained the effect on cholesterol and SREBP2 activation (Figs. 1f, g and 5b–e) and the conjugates synergized with simvastatin (Fig. 6c, d). These new developments support the idea that the overall cytotoxicity of a given S2L-based drug conjugate represents the sum of activities of the S2L portion of the molecule and the respective covalently linked cargo component.

In conclusion, our study provides additional insight into the mechanism of S2L-mediated cytotoxicity. S2L exposure triggered an increase in intracellular free cholesterol which causes ER stress which in turn initiates the unfolded protein response and activates SREBP2 as an adaptive mechanism. S2L combined with regimens that block this adaptive mechanism further sensitizes toward more efficient cell death. S2L drug conjugates retain the abilities of their parent ligands. These observations pave the way for exploring strategies in which S2L-based drug conjugates will be combined with cholesterol-lowering compounds, such as the FDA-approved simvastatin, or ER stress inducers to perform high-efficient cancer therapy across a wide spectrum of human malignancies.

### Supplementary information

Raw data of lipidomics analysis in vitro and in vivo.

Gene set enrichment analysis of RNAseq data from SYO1 cells treated with C0, C6, or C10 for 2 hours.

Representative images of western blots across all cell lines.

Supplemental Materials

## Data Availability

All data generated or analyzed during this study are included in this published article and its Supplementary Information files. The RNAseq data generated in this study have been deposited in NCBI’s Gene Expression Omnibus and are accessible through GEO Series accession number GSE260557 (https://www.ncbi.nlm.nih.gov/geo/query/acc.cgi?acc=GSE260557). Further information and requests for resources and reagents should be directed to and will be fulfilled by the corresponding authors, DS (spitzerd@musc.edu) or WGH (hawkinwi@musc.edu).
