# Supplementary figures and images for: Cytotoxic sigma-2 ligands trigger cancer cell death via cholesterol-induced-ER-stress
Source: Cell Death Dis. 2024 May 2;15(5):309. doi: 10.1038/s41419-024-06693-8 (PMC11066049; doi:10.1038/s41419-024-06693-8)

Time course

SREBP2

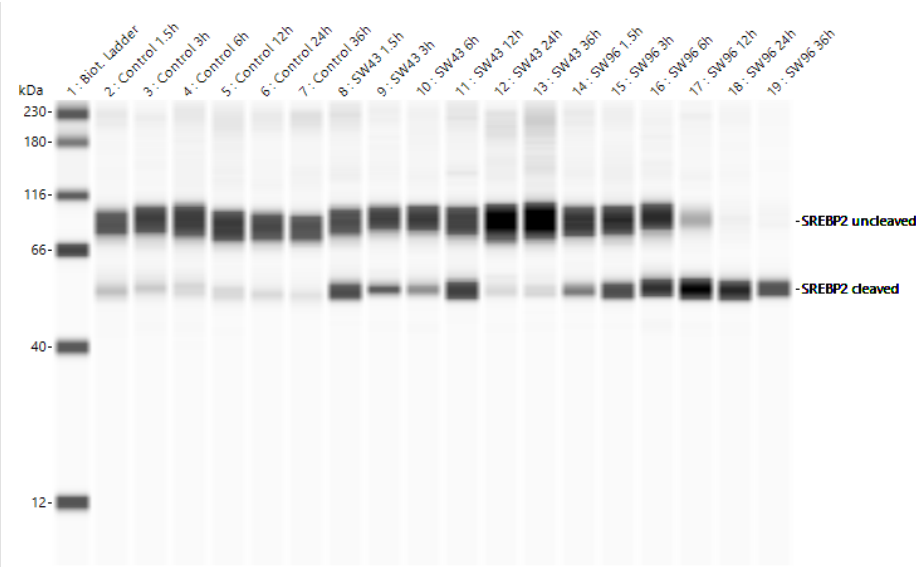

24h

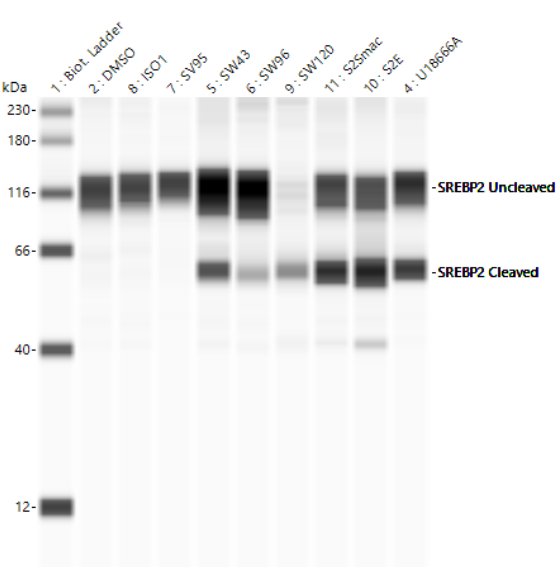

Time course

LDLR

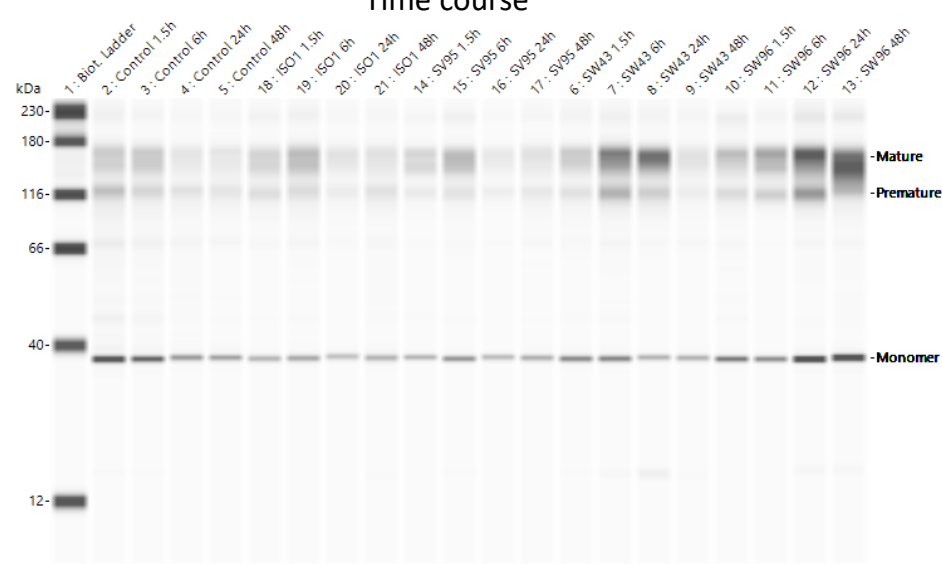

24h

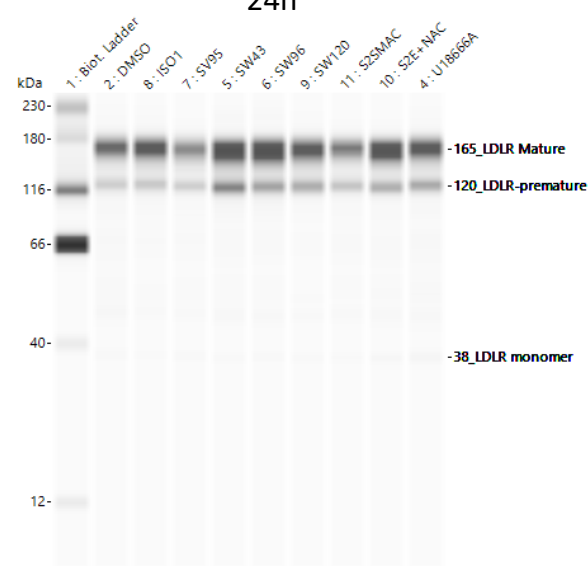

Additional file 9b. HPAC

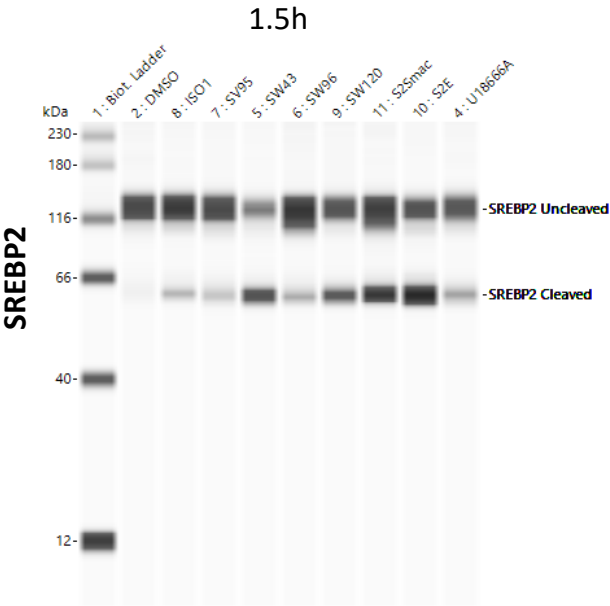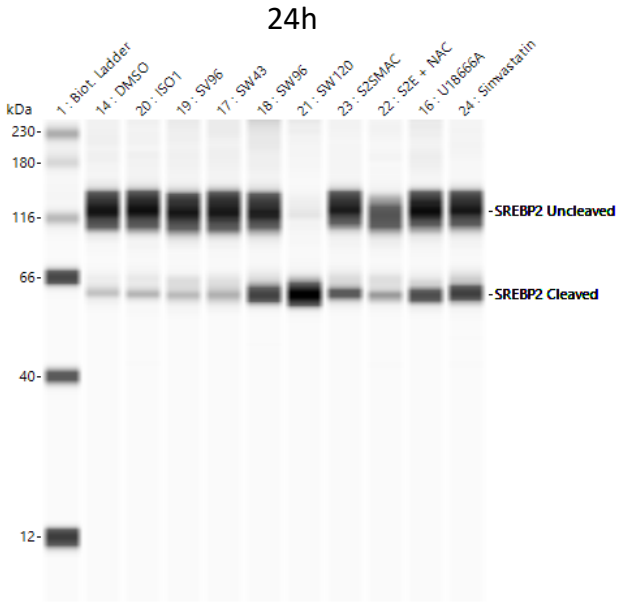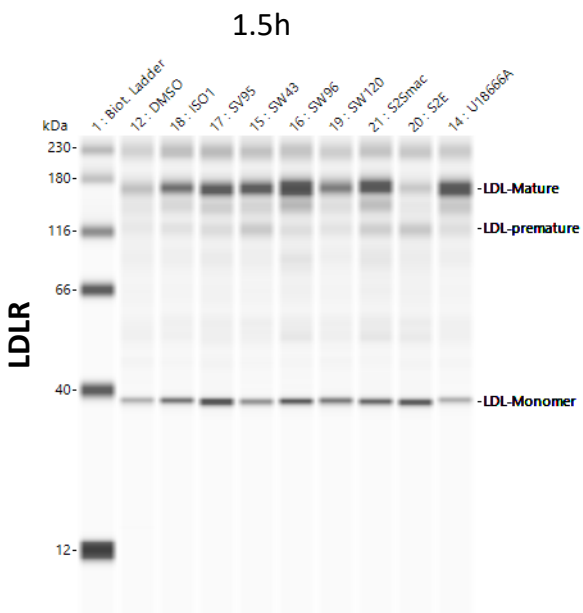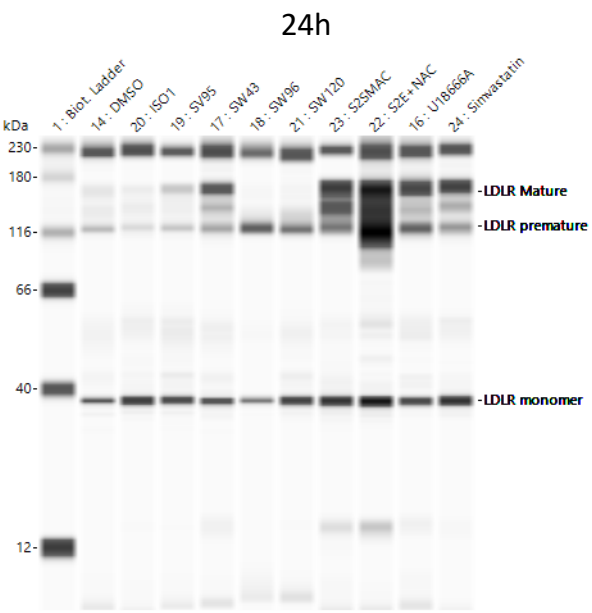

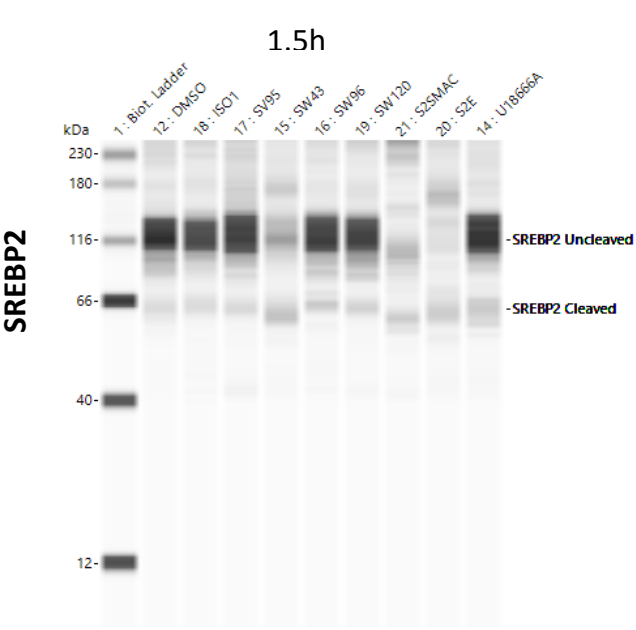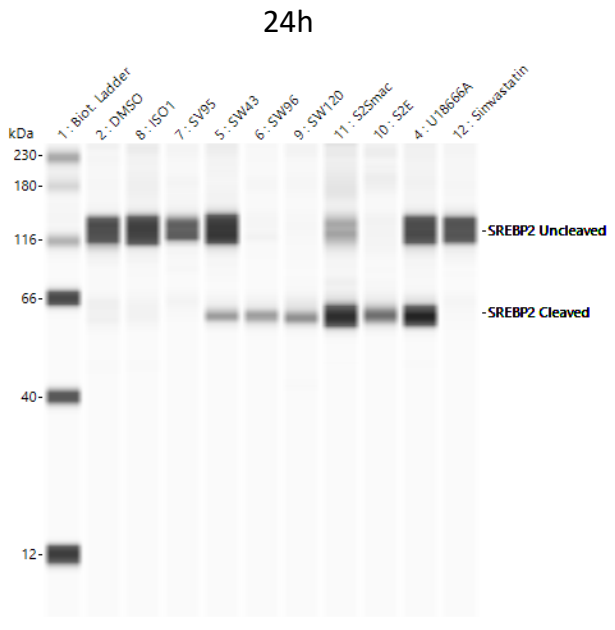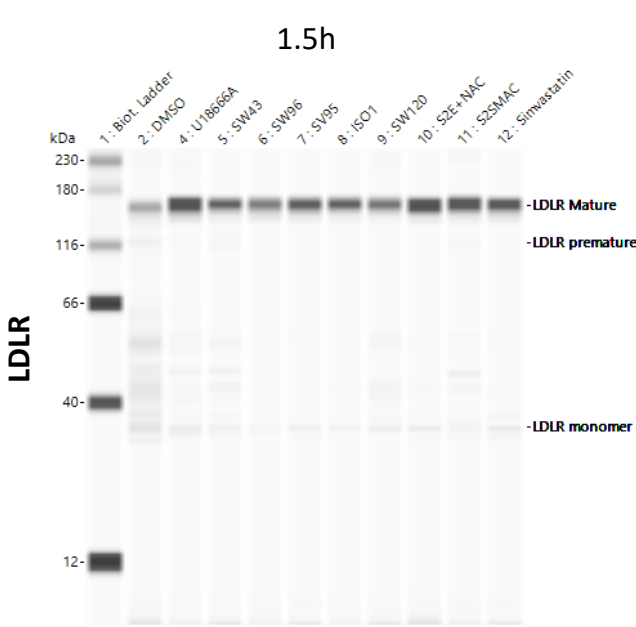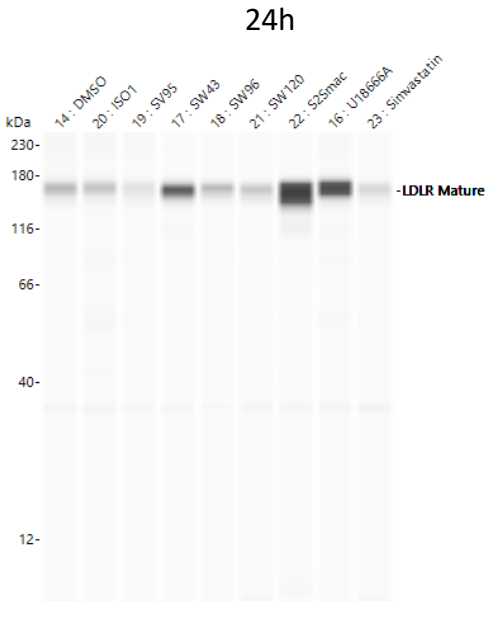

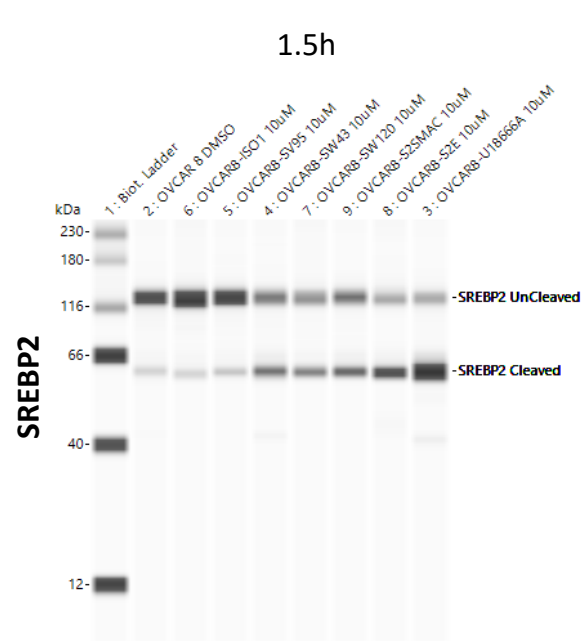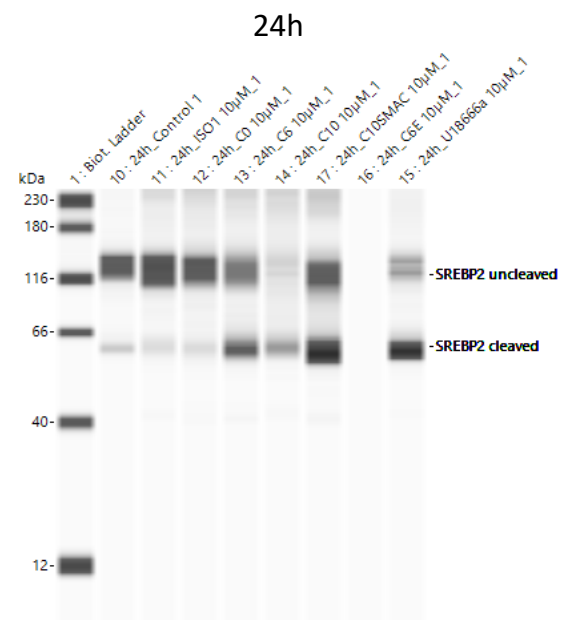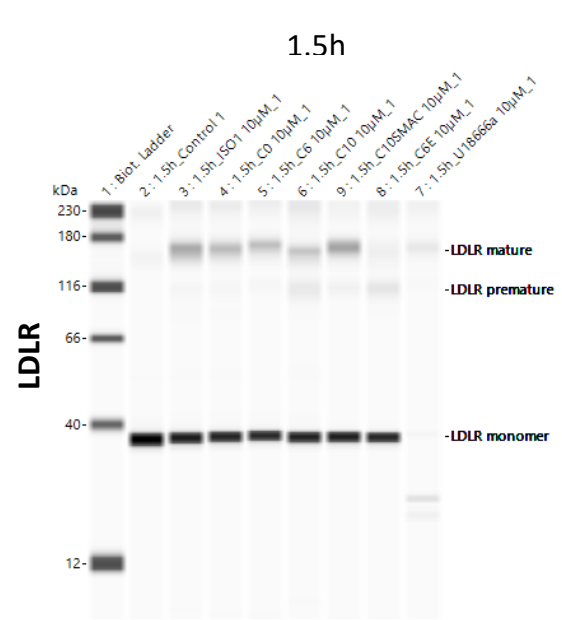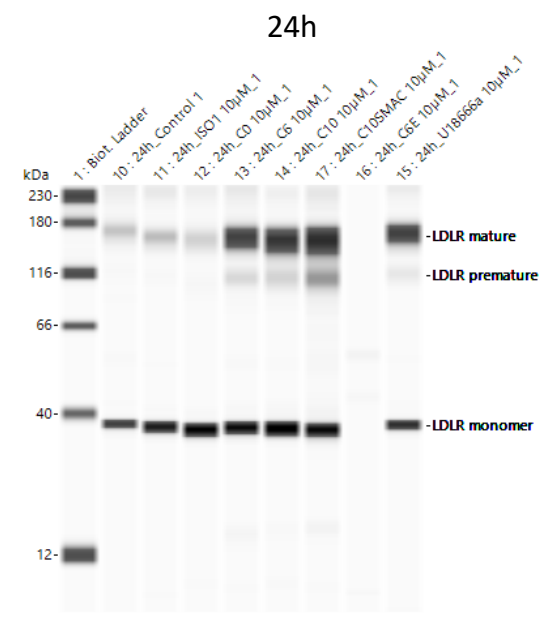

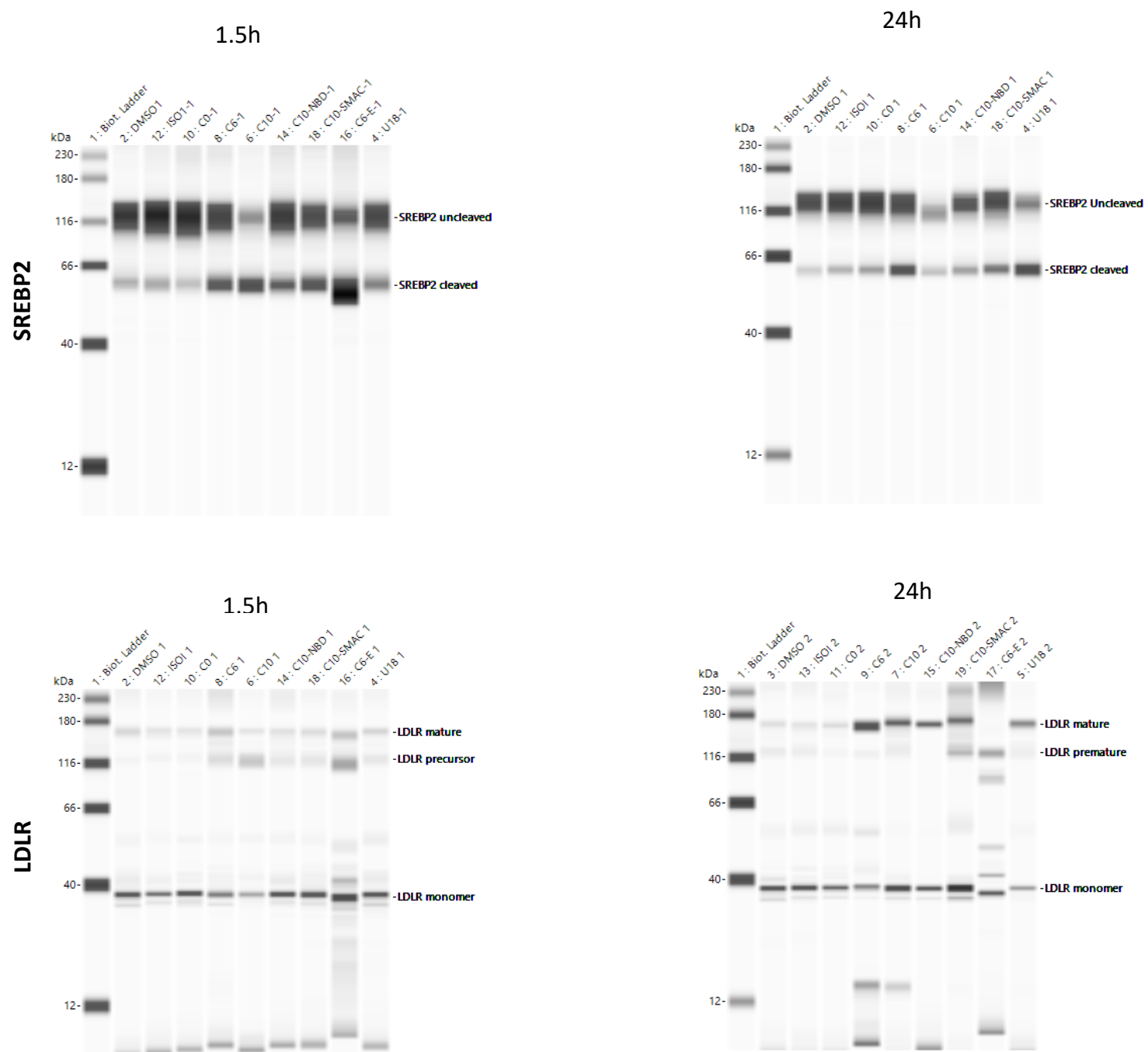

Supplement: Supplementary file 3 — Representative images of western blots across all cell lines. [file 41419_2024_6693_MOESM3_ESM.pdf]
