# Supplementary material for: Cytotoxic sigma-2 ligands trigger cancer cell death via cholesterol-induced-ER-stress
Source: Cell Death Dis. 2024 May 2;15(5):309. doi: 10.1038/s41419-024-06693-8 (PMC11066049; doi:10.1038/s41419-024-06693-8)
Supplement: Supplementary file 4 — Supplemental Materials [file 41419_2024_6693_MOESM4_ESM.pdf]

# Cytotoxic Sigma-2 Ligands Trigger Cancer Cell Death via Cholesterol-Induced-ER-Stress.

## Running title: S2L Trigger Cholesterol-Induced-ER-stress

**Authors**
Rony Takchi[1], Bethany C. Prudner[2], Qingqing Gong[1], Takaomi Hagi[1], Kenneth F. Newcomer[1], Linda X. Jin[1], Suwanna Vangveravong[1], Brian A. Van Tine[2,3,4], William G. Hawkins[1,4,*] , and Dirk Spitzer[1,4,*]

## Supplementary Material

**Additional file 1. Supplementary Materials and Methods**

**Additional file 3. S2L structures and affinities to S2R**

**Additional file 4. Cytotoxicity of S2L across cancer cells**.

Proliferation curves of pancreatic (AsPC-1, HPAC, MIAPaCa-2, KP2), ovarian (OVCAR-8), and synovial sarcoma (SYO-1) cell lines treated with various S2L for 24 or 72 hours. Data represents mean ± SD; n=3.

**Additional file 5. Subcellular localization of C10-NBD**

Representative confocal microscopy images of HPAC cells treated with 100 nM C10-NBD for 20 min and counterstained for lysosome, nucleus, mitochondria, and endoplasmic reticulum compartments. Scale bar equals 20 μm.

**Additional file 6. Cytotoxicity of U18666a compared to C10 across cancer cells** Proliferation curves of AsPC1, HPAC or SYO1 cell lines treated for 72 hours with U18666a or C10. In HPAC, IC50 was determined to be 30.1 ± 9.1 vs 18.5 ± 1.0 for U18666a vs C10.

**Additional file 7. Confocal images visualizing free cholesterol and cholesterol esters.**

**Additional file 10. Representative confocal image visualizing the effect of C10 over time in AsPC1.**

**Table S1: List of resources**

## Additional file 1: Supplementary Materials and Methods

### NucRed Cell line generation

Cell lines were transduced with IncuCyte NucLight Red lentivirus reagent (EF-1 Alpha promoter, Puromycin selection) according to their standard protocol. Briefly, cells were seeded and allowed to adhere for 24 h to reach approximately 30% confluence. NucLight reagent was diluted in media containing 8 μg/mL polybrene and added to cells for 48 h. Media was then replaced with fresh media containing 3 μg/mL puromycin. Red fluorescence was monitored and analyzed using on the Essen IncuCyte ZOOM system.

### Proliferation and cell-death assay

NucRed+ cells were seeded in 96-well plate at 5000-7000 cells per 100 µL media. At time of treatment, culture media was removed and replaced with phenol-free DMEM supplemented with 10% FBS, 2 mM GlutaMAX, and 50 nM Yoyo-1 Iodide (491/509). IncuCyte ZOOM time-lapse microscopy was used to monitor red nuclear fluorescence and green yoyo-1 fluorescence. After taking a baseline image, treatments of interest were added then plates were subsequently imaged every 2-hours for up to 72 hours. The images were analyzed using the IncuCyte image analysis software. Red and green object count/mm$^2$ , which quantified survival and cell-death signals respectively, were exported into Microsoft Excel. Because a baseline image was taken pretreatment, a corrected green table was calculated by subtracting the green signal at time 0 from all subsequent measurements. At any given time point *n* in a treatment time course (t=0 → t= *n*), the cell death signal was calculated using Equation 1:

$$Cell\ Death\ = \frac{Corrected\ Yoyo{-}1^{+}_{max_{t-0\ \rightarrow t=n}}}{(Corrected\ Yoyo{-}1^{+}_{max_{t-0\ \rightarrow t=n}} + NucRed^{+}_{t=n} - Overlay^{+}_{t=n})} \qquad \text{(Equation 1)}$$

Proliferation curves were calculated using Equation 2:

$$Proliferation = (1 - Death) = \frac{NucRed^{+}_{t=n} - Overlay^{+}_{t=n}}{(Corrected\ Yoyo{-}1^{+}_{max_{t-0 \rightarrow t=n}} + NucRed^{+}_{t=n} - Overlay^{+}_{t=n})}$$

(Equation 2)

As some treatments result in the loss of Yoyo-1$^{+}$ signal from long dead cell corpses, the death signal was computed with the maximum number of Yoyo-1$^{+}$ objects at any given time from the start of that experiment. Moreover, these equations separately consider the Yoyo-1$^{+}$& NucRed$^{+}$ double-positive cells (Overlay$^{+}$). Since a large number of the double-positive cells would lead to an underestimation of cell death, the Overlay$^{+}$ objects were subtracted from NucRed$^{+}$ objects. Area under the curve (AUC) values were computed from the cell death curves (or survival curves) over time using the trapezoidal method in GraphPad. The results are summarized as mean ± SEM. Non-linear regression curve fitting was used to calculate IC50 and EC50 values.

**Confocal microscopy time-lapse or Z-stack**

The Zeiss LSM 880 with airyscan confocal laser scanning microscope was used with a plan-apochromat x40 (NA 1.3) / x63 (NA1.4) oil objective.

To visualize subcellular localization of C10-NBD, cells were plated on 35 mm dish 1.5# glass bottom overnight. The samples were washed with PBS, then stained according to the recommended concentration. [LysoTracker® Deep Red (50 nM); MitoTracker™ Deep Red FM (50 nM); ER-Tracker™ Red (1 µM). To stain the nucleus, 2 drops/mL of NucBlue™ Live ReadyProbes™ Reagent. After incubating at 37°C (except NucBlue at room temp) for 30 minutes, the samples were washed twice with PBS, then allowed to incubate for 15 min at room temp in ProLong Live Antifade Reagent solution 1:100 in phenol free media. After adding 100nM C10-NBD, 30-minutes live time-lapse videos were recorded using the following lasers:

NucBlue (405 nm), NBD (458 nm), ER Tracker (561 nm), LysoTracker and Mitotracker Deep Red (633 nm).

To visualize intracellular distribution of free cholesterol with Filipin stain, cells were plated on 35 mm dish 1.5# glass bottom overnight, after which different treatments were added for another 24 hrs. The samples were stained with 50 nM LysoTracker® Deep Red, fixed with 4% paraformaldehyde solution, then stained with 5 mg/mL filipin stain. After washing, one drop of slowfade™ gold antifade mountant was added before sealing the sample with a coverslip and nail polish. Samples were stored in dark at 4°C. Z-stack images were taken using the following lasers: Filipin (405 nm), LysoTracker (633 nm). Zen Black 2.3 SP1 version 14.0.15.201 software was used to both capture the images and post-processes them to combine the multiple AiryScan 32-detector array images into de-convolved final images with high signal-to-noise ratio and resolution. Z-stack images were exported as .TIF, and filipin puncta were quantified using ImageJ.

**Additional file 3.** S2L structures and affinities to S2R.

## Benzamide analogs

**ISO1**

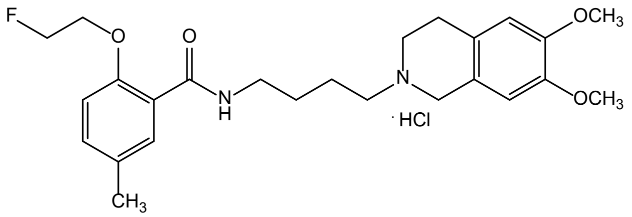

σ1 Ki (nM) = 330 ± 25
σ2 Ki (nM) = 7.0 ± 1.6
σ1/ σ2 = 48

## Azabicyclononane analogs

**C0**
(SV95)

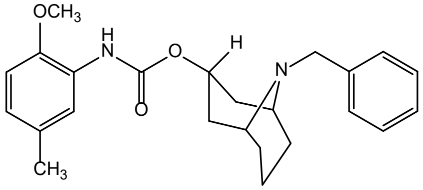

σ1 Ki (nM) = 92.5 ± 11.0
σ2 Ki (nM) = 3.1 ± 0.8
σ1/ σ2 = 30.0

Backbone

**C6**
(SV119)

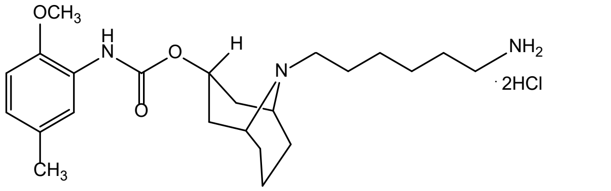

σ1 Ki (nM) = 1418 ± 439
σ2 Ki (nM) = 5.2 ± 0.8
σ1/ σ2 = 273

Conjugate

**C6-Erastin**
(ACXT3102)

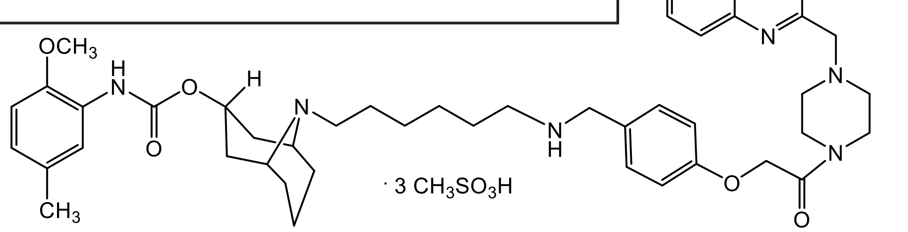

σ1 Ki (nM) = 2893 ± 369
σ2 Ki (nM) = 1537 ± 65
σ1/ σ2 = 1.9

Backbone

**C10**
(SW43)

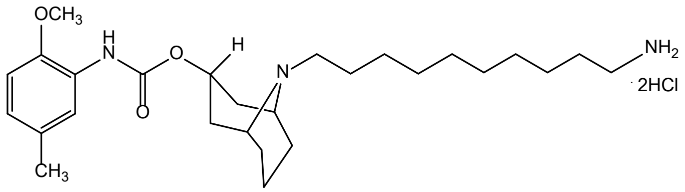

σ1 Ki (nM) = 134 ± 11.9
σ2 Ki (nM) = 7.1 ± 1.3
σ1/ σ2 = 19.0

Conjugates

**C10-NBD**
(SW120)

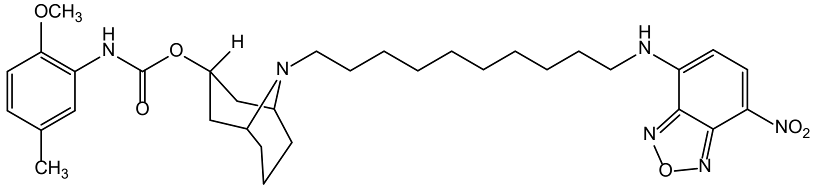

σ1 Ki (nM) = 449 ± 29
σ2 Ki (nM) = 11.0 ± 2.4
σ1/ σ2 = 40.9

**C10-capped**
(SW-V-96)

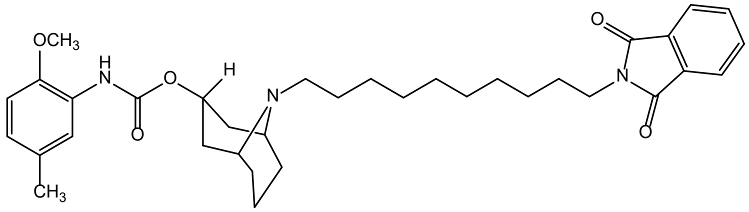

σ1 Ki (nM) = 7671 ± 609
σ2 Ki (nM) = 896 ± 20.2
σ1/ σ2 = 8.6

**C10-SMAC**
(SW-IV-134)

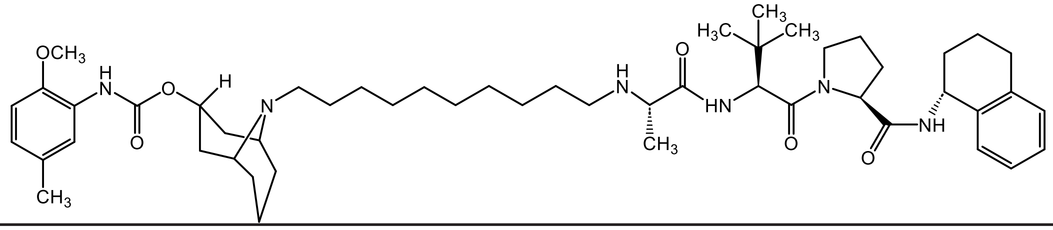

σ1 Ki (nM) = 5737 ± 476
σ2 Ki (nM) = 22.6 ± 1.8
σ1/ σ2 = 253.8

## Additional file 4

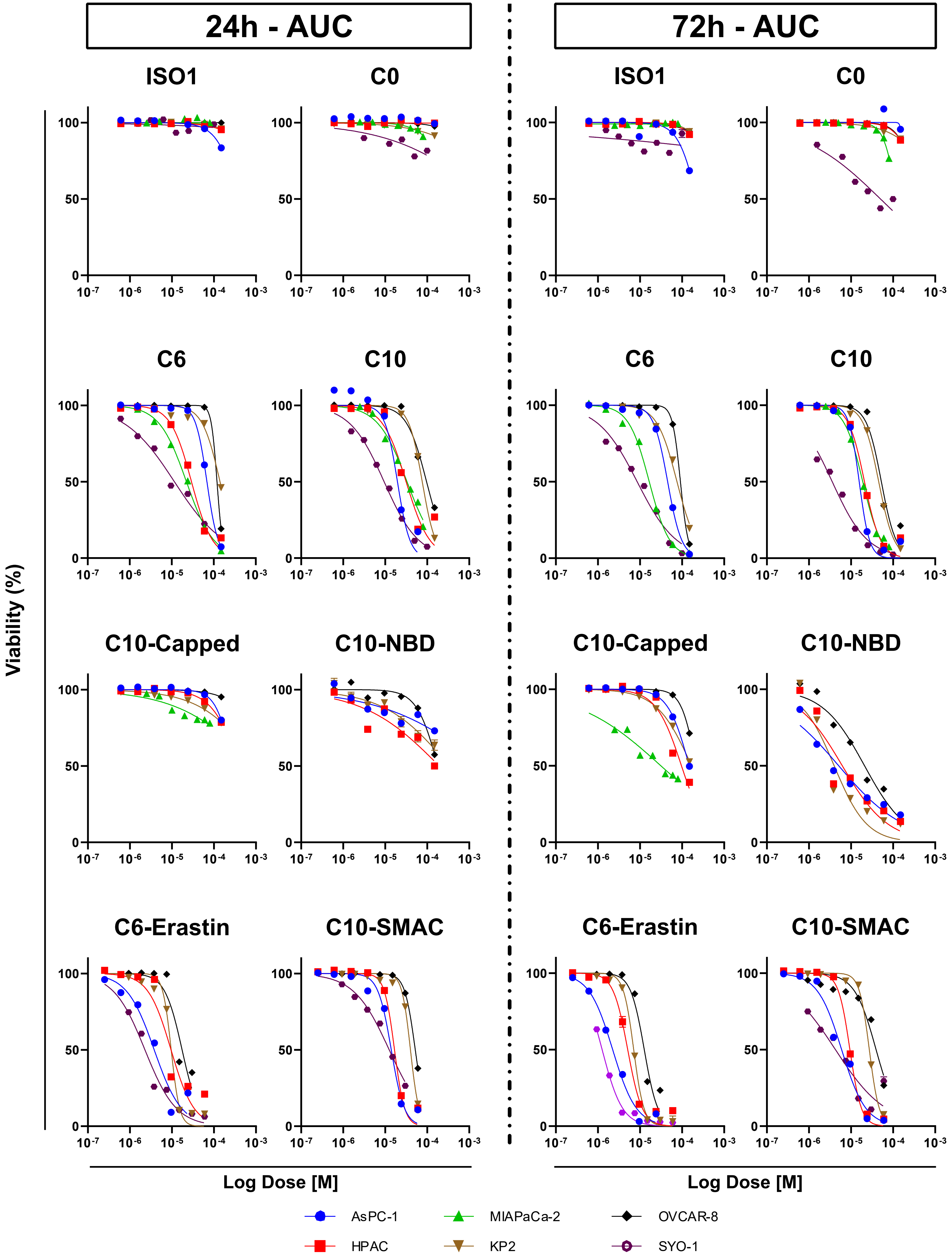

Additional file 5. Subcellular localization of C10-NBD

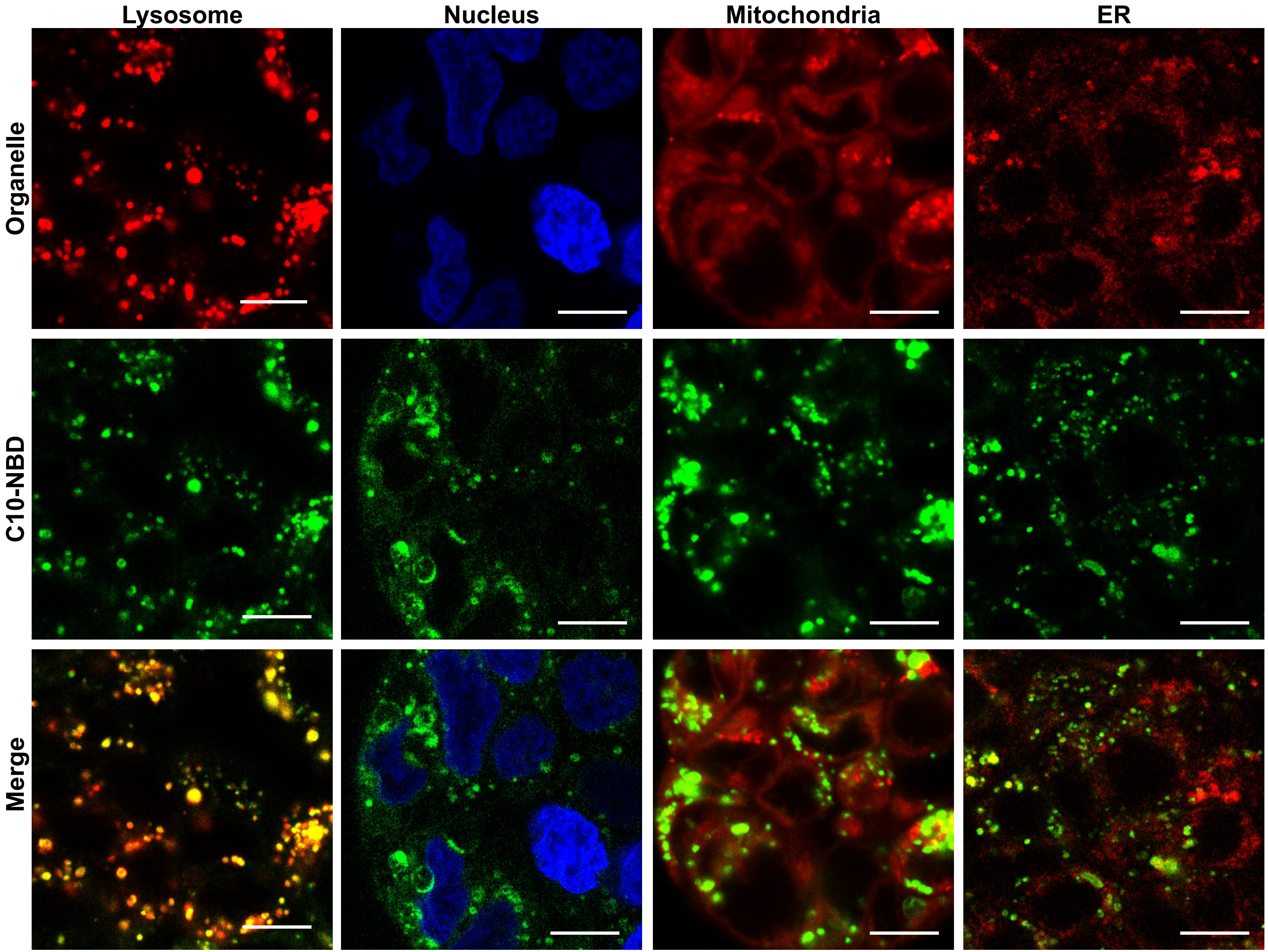

Additional file 6

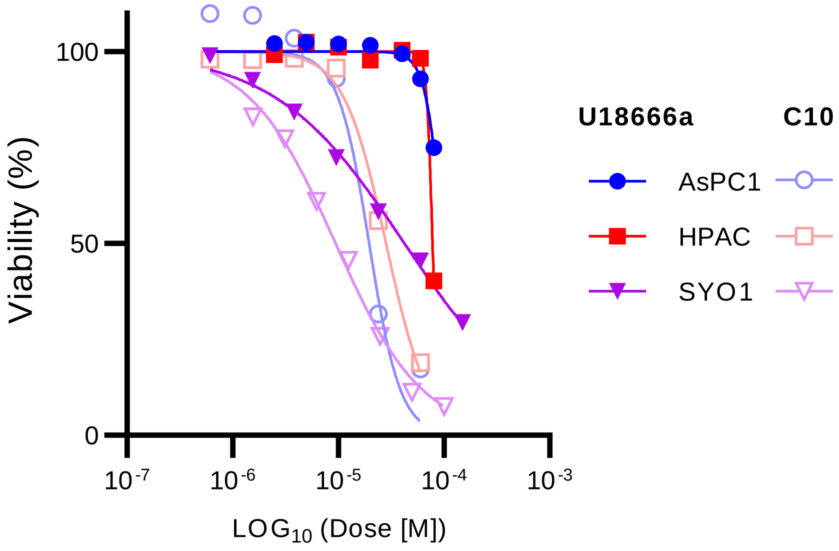

Additional file 7

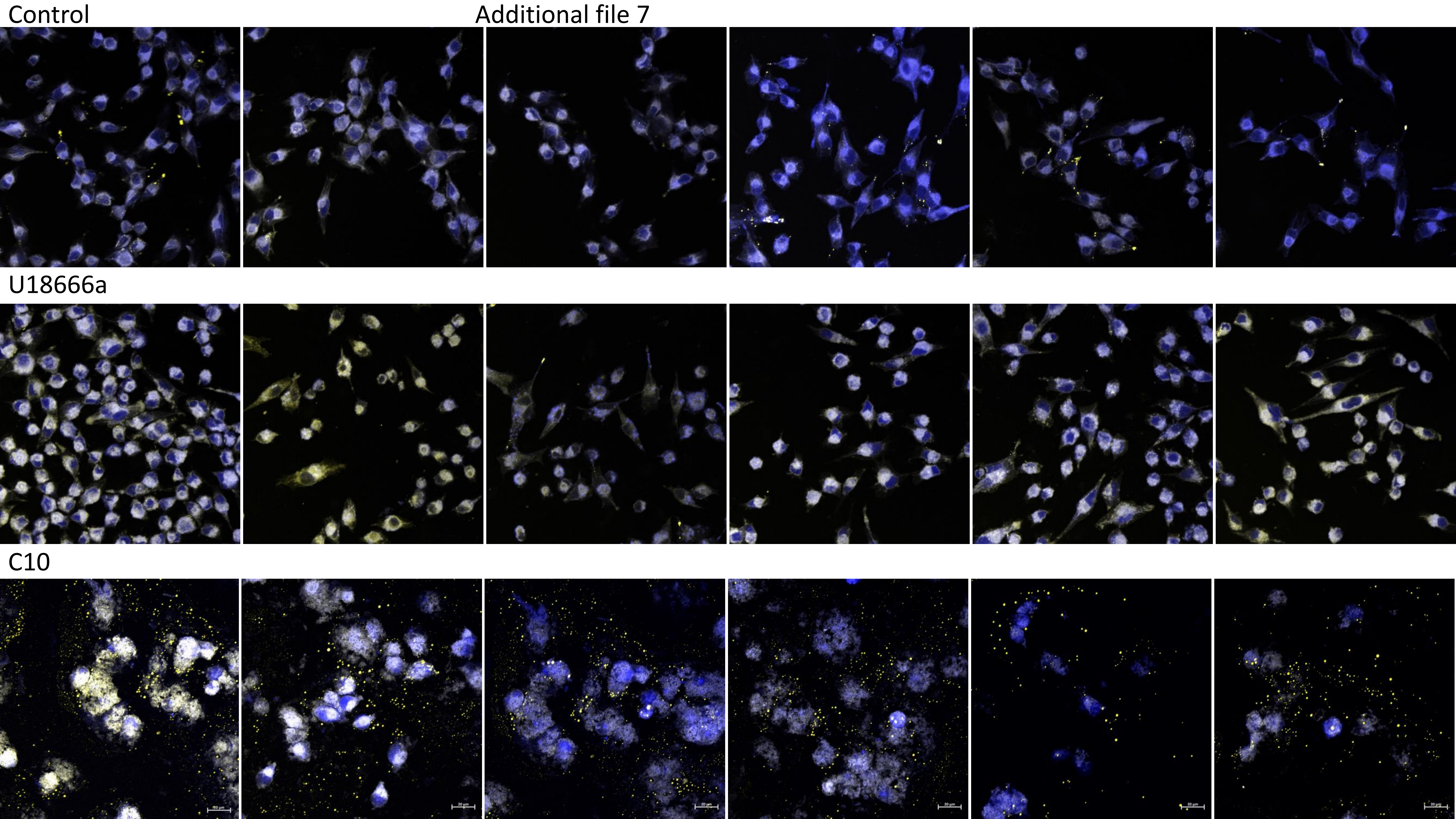

Additional file 10

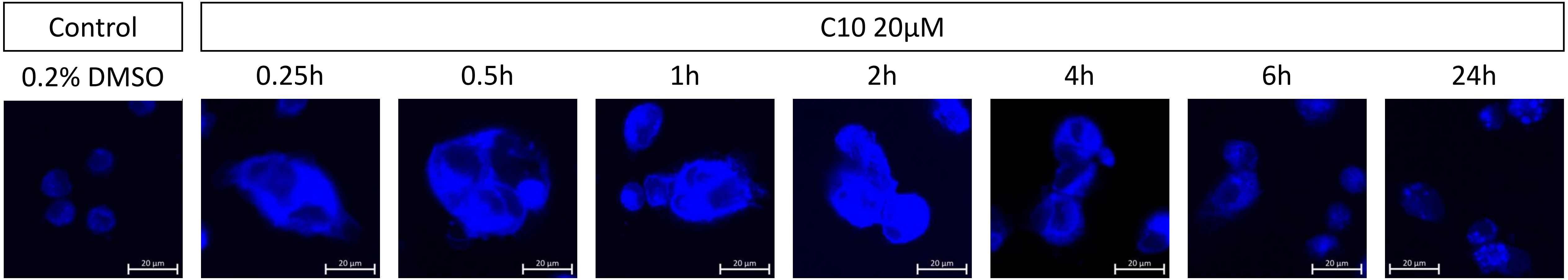

**Table S1:** List of resources

| Reagent or Resource | Source | Identifier |
|---|---|---|
| **Antibodies** | | |
| Anti-SREBP2 | Active Motif | Cat# 39941; RRID:AB_2793400 |
| Anti-LDLR | Abnova | Cat# PAB8804, RRID:AB_1676510 |
| Anti-rabbit | Protein Simple | Cat# DM-001 |
| Anti-mouse | Protein Simple | Cat# DM-002 |
| Total protein | Protein Simple | Cat# DM-TP01 |
| **Bacterial and virus strains** | | |
| Incucyte® NucLight Red Lentivirus Reagent (EF-1 Alpha Promoter, Puromycin selection) | Sartorius | Cat# 4476 |
| **Media, Chemicals, peptides, and recombinant proteins** | | |
| RPMI 1640 | Gibco | Cat# 11875-085 |
| DMEM | Gibco | Cat# 11965-084 |
| DMEM-phenol free | Gibco | Cat# 21063-029 |
| Ham's F-12 Nutrient Mix | Gibco | Cat# 11765-054 |
| Fetal Bovine Serum Premium | Atlanta biologicals | Cat# S11150 Lot# D19043 |
| Antibiotic-Antimycotic | Thermo fisher | Cat# 15240112 |
| Prophylactic Plasmocin | InvivoGen | Cat# at-mpp |
| Polybrene | EMD Millipore | Cat# #TR-1003-G |
| Puromycin | InvivoGen | Cat# ant-pr-1 |
| GlutaMAX | Gibco | Cat# 35050-061 |
| Yoyo-1 Iodide (491/509) | Invitrogen | Cat# Y3601 |
| Pitstop 2 | Abcam | Cat# ab120687 |
| Cell stripper | Corning | Cat# 25-056-CI |
| LysoTracker® Deep Red | Thermo fisher | Cat# L12492 |
| MitoTracker™ Deep Red FM | Thermo fisher | Cat# M22426 |
| ER-Tracker™ Red | Thermo fisher | Cat# E34250 |
| NucBlue™ Live ReadyProbes™ Reagent | Thermo fisher | Cat# R37605 |
| ProLong Live Antifade Reagent | Thermo fisher | Cat# P36974 |
| Matrigel | Corning | Cat# 356234 |
| Kolliphor EL | Sigma Aldrich | Cat# C5135 |
| Molecular grade water | Corning | Cat# 46-000-CM |
| Filipin stain | Sigma Aldrich | Cat# F9765 |
| SlowFade™ Gold antifade mountant | Thermo fisher | Cat# S36937 |
| RIPA buffer 10X | Cell Signaling | Cat# 9806S |
| Halt protease inhibitor 100X | Thermo fisher | Cat# 78443 |
| TRIzol™ Reagent | Invitrogen | Cat# 15596018 |

| | | |
|---|---|---|
| **Critical commercial assays** | | |
| Pierce™ BCA Protein Assay | Thermo fisher | Cat# 23224, #23228 |
| SMARTer® Ultra® Low Input RNA for Illumina® Sequencing - HV | Takara | Cat# 634828 |
| CellTiter-Glo® 2.0 Cell Viability Assay | Promega | Cat# G924C Lot# 479866 |
| QuantiTech Reverse Transcription Kit | Qiagen | Cat# 205311 |
| iQ™ SYBR® Green Supermix | BioRad | Cat# 1708880 |
| **Experimental models: cell lines** | | |
| AsPC-1 | ATCC | Cat# CRL-1682; RRID:CVCL_0152 |
| HPAC | ATCC | Cat# CRL-2119; RRID:CVCL_3517 |
| MIAPaCa-2 | ATCC | Cat# CRL-1420; RRID:CVCL_0428 |
| KP2 (mouse) | Derived from pancreatic cancer tumor tissue obtained from p48-CRE/LSL-KrasG12D/p53flox/+ mice (backcrossed C57BL/6, n = 6) | |
| OVCAR-8 | Gift from Dr. Katherine C. Fuh | RRID:CVCL_1629 |
| SYO-1 | Gift from Dr. Brian A. Van Tine | RRID:CVCL_7146 |
| **Experimental models: organisms/strains** | | |
| Mouse: NCI C56BL/6NCr (female, 4-6 weeks old, inbred) | Charles River | Cat# 556 |
| **Tools, software, and algorithms** | | |
| Incucyte Zoom System | Sartorius | RRID:SCR_019874 |
| GraphPad Prism v9.4.1 | GraphPad | RRID:SCR_002798 |
| Excel | Microsoft | RRID:SCR_016137 |
| Compusyn 1.0 | ComboSyn, Inc. | RRID:SCR_022931 |
| Compass for Simple Western | Protein Simple | RRID:SCR_022930 |
| FACSCalibur Flow Cytometry System | BD life sciences | RRID:SCR_000401 |
| FlowJo_v10.7.1 | BD life sciences | RRID:SCR_008520 |
| LSM 880 with Airyscan Confocal Laser Scanning Microscope | Zeiss | RRID:SCR_020925 |
| Zen Black 2.3 SP1 version 14.0.15.201 software | Zeiss | RRID:SCR_018163 |
| ImageJ v1.52 | Fiji | RRID:SCR_003070 |
| Flow software | Partek | RRID:SCR_011860 |
| Nanodrop 2000 spectrophotometer | Thermo Fisher Scientific | RRID:SCR_018042 |
| TissueLyser II | Qiagen | RRID:SCR_018623 |

| | | |
|---|---|---|
| Bio-Rad CFX96 Real-Time PCR Detection System | BioRad | RRID:SCR_018064 |
| Online sample size calculator | Clinic Calc. | https://clincalc.com/stats/samplesize.aspx |
| Biorender | Biorender | RRID:SCR_018361 |
| Partek Genomics Suite | Partek | RRID:SCR_011860 |
| Gene Set Enrichment Analysis software v4.3.3 | Broad Institute | RRID:SCR_003199 |
| Cytoscape v3.10.1 | Cytoscape International Consortium | RRID:SCR_003032 |
| AutoAnnotate v1.4.1 | Cytoscape App | http://www.baderlab.org/Software/AutoAnnotate |
| EnrichmentMap v3.3.5 | Cytoscape App | https://enrichmentmap.readthedocs.io/en/latest/ |
| **Deposited data** | | |
| RNA-Seq of SYO1 cells treated for 2 hours with 10µM of various sigma-2 ligands (control, C0, C6, C10) in triplicates. | This paper | GEO:GSE260557 |
| Broad Institute Hallmark [50 gene sets] | MSigDB 2023.2.Hs | https://www.gsea-msigdb.org/gsea/msigdb/human/genesets.jsp?collection=H |
| Broad Institute Gene Ontology Biological Process (GO:BP) [7,647 gene sets] | MSigDB C5.go.bp.v2023.2.Hs | https://www.gsea-msigdb.org/gsea/msigdb/human/genesets.jsp?collection=GO:BP |

**Primers**

| Gene | Species | Forward Primer | Reverse Primer | Size, bp |
|---|---|---|---|---|
| *RPLO* | Human | GGCGACCTGGAAGTCCAACT | CCATCAGCACCACAGCCTTC | 143 |
| *SREBP2* | Human | TAACCCCCTGACTTCCCTGC | GCTGCAAAATCTCCTCTGGC | 294 |
| *LDLR* | Human | CAGCTACCCCTCGAGACAGA | CACTGTCCGAAGCCTGTTCT | 202 |
| *NPC1* | Human | CTTACTGGGAGCCACTCACG | CGACCGACCCTTAGACACAG | 185 |
| *HMGCR* | Human | AGTGAGATCTGGAGGATCCAAG | AGGATGGCTATGCATCGTGT | 258 |
| *MVK* | Human | GTACCTCGTGCTGGAAGAGC | GCTTGAGGAGTGTGATGCCA | 167 |
| *MVD* | Human | GTCATCAAGTACTGGGGCAAG | TTCAGCCAAATCCGGTCCTC | 146 |
| *NSDHL* | Human | GCGCCTACGGACGGAAAAGA | CACTGTGCATCTCTTGGCCT | 183 |
| *ATF4* | Human | CTTGATGTCCCCCTTCGACC | CGGAGAAGGCATCCTCCTTG | 197 |
| *CHAC1* | Human | TTGAAGATCATGAGGGCTGC | GGTATCGTAGCCACCAAGCA | 116 |
| *DDIT3* | Human | ACCTGAGGAGAGAGTGTTCAA | GGCTGGAACAAGCTCCATGT | 200 |
